# Extracellular LCN2 Binding to 24p3R in Astrocytes Impedes α‐Synuclein Endocytosis in Parkinson's Disease

**DOI:** 10.1002/advs.202501694

**Published:** 2025-07-21

**Authors:** Ying‐Ying Jiao, Tian Tian, Zhu Zhu, Lei Cao, Yang Liu, Rui‐An Wang, Zhi‐Yong Zhou, Cong Wang, Ke‐Zhong Zhang, Xiao Lu, Wen‐Wei Yun, Ying‐Mei Lu, Gang Hu, Ren‐Hong Du, Ming Lu

**Affiliations:** ^1^ Jiangsu Key Laboratory of Neurodegeneration, Department of Pharmacology Nanjing Medical University Nanjing 211166 P. R. China; ^2^ Changzhou Second People's Hospital, Changzhou Medical Center Nanjing Medical University Changzhou 213000 P. R. China; ^3^ State Key Laboratory of Pharmaceutical Biotechnology Nanjing University Nanjing 210023 P. R. China; ^4^ Department of Pharmacology Nanjing University of Chinese Medicine Nanjing 210023 P. R. China; ^5^ Department of Neurology The First Affiliated Hospital of Nanjing Medical University Nanjing P. R. China; ^6^ Department of Rehabilitation Medicine The First Affiliated Hospital of Nanjing Medical University Nanjing 210029 P. R. China; ^7^ Department of Physiology, School of Basic Medical Sciences Nanjing Medical University Nanjing 211166 P. R. China

**Keywords:** 24p3R, astrocytes, lipocalin‐2, Parkinson's disease, α‐synuclein

## Abstract

The spread or transmission of pathologic α‐synuclein (α‐Syn) is emerging as potentially important driver of Parkinson's disease (PD) pathogenesis. Emerging evidence suggests that astrocytes play an important role in uptake/clearance of extracellular α‐Syn. However, underlying mechanisms and molecular entities responsible for uptake/clearance of extracellular α‐Syn by astrocytes are not known. Here, it is shown that lipocalin‐2 (LCN2) is upregulated in astrocytes of MPTP‐treated mice by RNA‐Seq analysis and positively correlates with pathologic α‐Syn level in α‐Syn PFF model. Strikingly, deletion of astrocytic LCN2 significantly prevents the pathologic α‐Syn accumulation and neurodegeneration. Moreover, 24p3R as a crucial receptor of α‐Syn uptake by astrocytes is identified, as well as an important mediator of α‐Syn spread in the brain. 24p3R specifically binds to α‐Syn and then mediates α‐Syn uptake. LCN2 prevents astrocytic uptake of α‐Syn by impeding the binding of 24p3R and α‐Syn. The identification of LCN2/24p3R as a key regulator of α‐Syn by astrocytes provides a new target for the treatment of PD and related α‐synucleinopathies.

## Graphical abstract

24p3R is an astrocytic receptor for α‐Syn uptake. When LCN2 is secreted from astrocytes, it binds to its receptor 24p3R in astrocytes and this binding impedes the α‐Syn PFF binding to 24p3R. This in turn inhibits 24p3R‐mediated α‐Syn PFF uptake and reduces the clearance of extracellular α‐Syn by astrocytes, which contributes to α‐Syn spread and neurodegeneration.

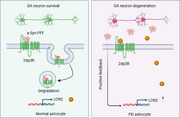

## 1 Introduction

Parkinson's disease (PD) is the second most common neurodegenerative disorder and is characterized by the accumulation of α‐synuclein (α‐Syn) in Lewy bodies and neurites.^[^ ^1^ ^]^ Accumulating evidence suggests that α‐Syn can be released from degenerating neurons during cell stress, leading to a higher concentration of α‐Syn aggregates in the extracellular space.^[^ ^2^ ^]^ These secreted extracellular α‐Syn could initiate the spreading of toxic forms of α‐Syn between neurons.^[^ ^3^ ^]^ Therefore, approaches that can promote the clearance of extracellular α‐Syn aggregates might be beneficial to prevent cell‐to‐cell pathologic α‐Syn propagation and the progression of the α‐Syn‐related diseases.

Although major brain cell types can uptake disease‐associated α‐Syn aggregates, glial cells appear to be the most efficient scavengers.^[^ ^4^ ^]^ Microglia, as professional phagocytes residing in the brain, can internalize and degrade α‐Syn fibrils, thereby being a major player in the cell‐to‐cell spreading and clearing of α‐Syn.^[^ ^5^ ^]^ Interestingly, astrocytes have been identified as key players in the clearance of extracellular α‐Syn.^[^ ^6^ ^]^ Toxic species of α‐Syn can be efficiently internalized by astrocytes and degraded via the lysosomal pathway at a higher rate than other cells.^[^ ^7^ ^]^ Therefore, astrocytes are considered as a particularly promising cell type that can be targeted in the development of novel therapeutics to improve α‐Syn clearance. However, underlying molecular mechanisms responsible for uptake of extracellular α‐Syn by astrocytes remains unclear.

Lipocalin‐2 (LCN2) is a secreted glycoprotein belonging to the lipocalin superfamily and has been recognized as a modulatory factor for diverse cellular phenotypes in the central nervous system (CNS), such as cell migration, cell survival, cell senescence, and inflammatory responses.^[^ ^8^ ^]^ Upregulated LCN2 is also involved in a variety of CNS disorders, including acute brain injuries such as ischemic stroke, anxiety, traumatic brain injury, and spinal cord injury, as well as age‐related brain diseases such as Alzheimer's disease and vascular dementia.^[^ ^9^ ^]^ 24p3R, the currently known cell surface receptor of LCN2, are reported to be expressed in glial cells, epithelial cells, and neurons.^[^ ^10^ ^]^ The binding of 24p3R to LCN2 mediates iron endocytosis.^[^ ^11^ ^]^ However, the involvement of LCN2/24p3R in uptake/clearance of extracellular α‐Syn by astrocytes has not been clarified.

In the present study, we identify 24p3R as an astrocytic receptor for α‐Syn uptake. 24p3R binding to α‐Syn mediates α‐Syn uptake. We further recognize that 153–183 residues of 24p3R are critical for α‐Syn binding and uptake. Lack of astrocytic 24p3R substantially accelerates the spread of pathologic α‐Syn and PD progression. LCN2 promotes pathological α‐Syn transmission and toxicity by impeding the binding of 24p3R and α‐Syn. Our study deepens the understanding of the molecular mechanism for uptake/clearance of extracellular α‐Syn by astrocytes, and sheds light on the development of disease modifying therapy for PD and aged‐related α‐synucleinopathies.

## 2 Results

### 2.1 LCN2 is Increased in Astrocytes of MPTP Model and α‐Syn PFF Model

**Figure 1:**
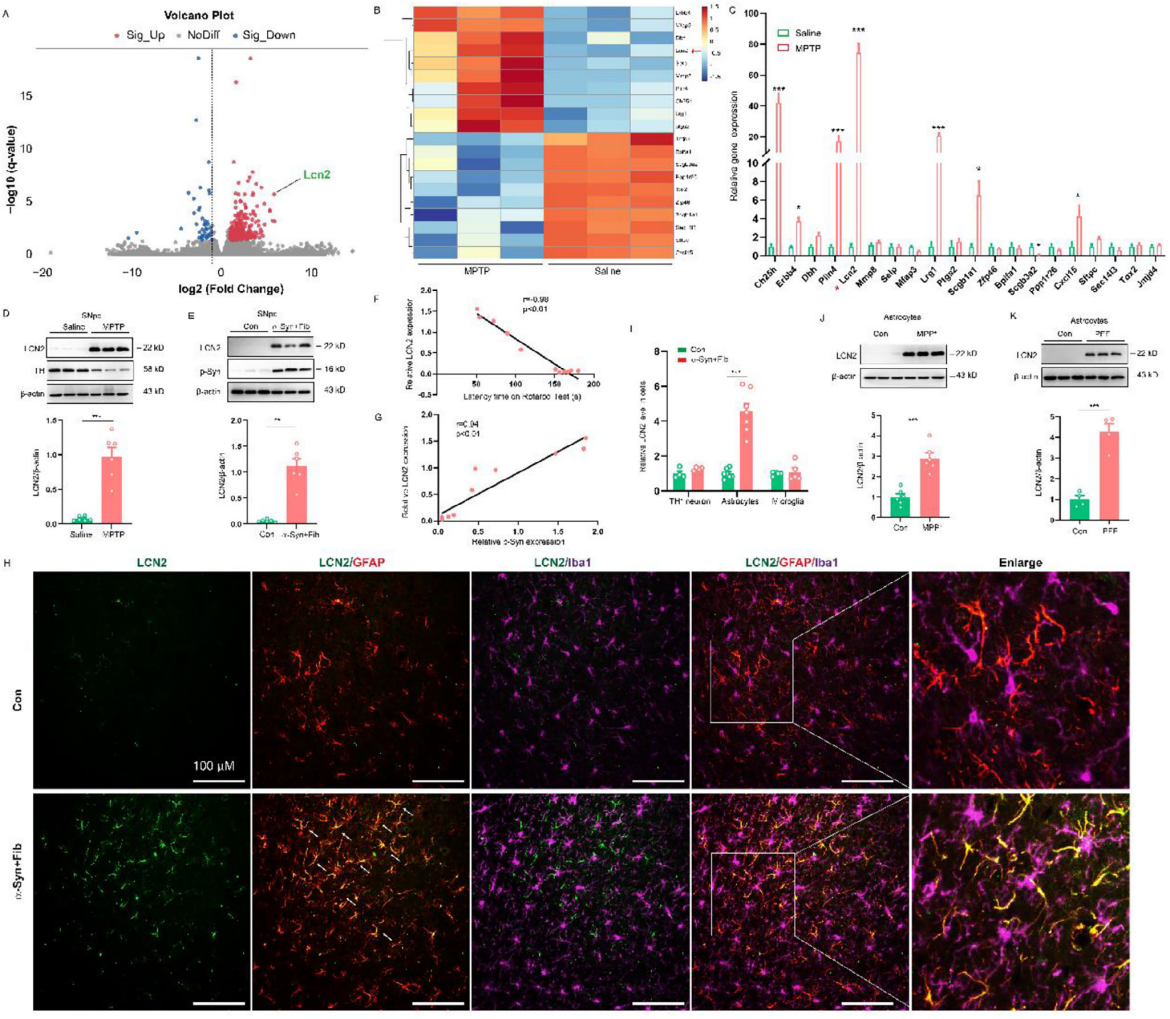
LCN2 is increased in astrocytes of MPTP model and α‐Syn PFF model. A) Volcano plot of downregulated (down) or upregulated (up) genes in the SNpc of MPTP‐treated mice compared to control mice by RNA‐seq analysis (n = 3 animals for each group). B) Heatmap of top 10 upregulated and 10 downregulated genes as indicated by RNA‐seq analysis. C) qPCR analysis measuring the mRNA levels of top 20 genes as indicated in the SNpc of MPTP‐treated mice (n = 6 animals for each group). D‐E) Representative immunoblots of relative expression of LCN2 in the SNpc of MPTP‐treated mice (D, n = 6 animals for each group) and α‐Syn PFF‐treated mice (E, n = 6 animals for each group). F) The LCN2 level is negatively correlated with the latency time on the rotarod test in α‐Syn PFF‐treated mice. G) The LCN2 expression is positively correlated with pathologic p‐α‐Syn level in the SNpc of α‐Syn PFF‐treated mice. H) LCN2 immunohistochemical signal in astrocytes and microglia from the SNpc of α‐Syn PFF‐treated mice after tyramide signal amplification. I) Quantification of the LCN2 level in TH /GFAP /Iba1 positive cells in α‐Syn PFF‐treated mice (n = 4–7 animals for each group). J‐K) Representative immunoblots of relative expression of LCN2 in astrocytes treated with MPP^+^ (J, six independent experiments) or α‐Syn PFF (K, four independent experiments) for 48 h. The data shown are the mean ± SEM. Unpaired t test was used (C–E, I–K) and correlation was analyzed by Pearson's correlation coefficient (F‐G). ^*^p < 0.05, ^**^p < 0.01, ^***^p < 0.001.

To uncover the pathological changes in PD, RNA‐seq was performed with total RNA extracted from the substantia nigra pars compacta (SNpc) of MPTP‐treated mice and control mice. 271 genes were upregulated and 53 genes were downregulated in the SNpc of MPTP‐treated mice compared to control mice (**Figure** **1A**, Table S1, Supporting Information). The RNA‐seq results were confirmed by qPCR with selected the TOP 10 downregulated genes and 10 upregulated genes in the SNpc. Among the above RNA‐seq‐identified differentially expressed genes, LCN2 was the most significantly upregulated gene in MPTP‐treated mice by qPCR (Figure 1B,C). Consistent with the mRNA assay, the protein expression of LCN2 was markedly augmented in the SNpc of both MPTP‐treated mice and α‐Syn PFF‐injected mice (Figure 1D,E). Importantly, the LCN2 expression was positively correlated with pathologic α‐Syn level and negatively correlated with motor function in α‐Syn PFF‐injected mice (Figure 1F,G). Subsequently, Immunostaining further confirmed that expression of LCN2 was evidently increased in MPTP‐treated mice and α‐Syn PFF‐ injected mice, mainly in GFAP^+^ astrocytes from the SNpc (Figure 1H,I; Figures S1 and S2, Supporting Information). Meanwhile, we repeated the in vivo model in primary culture astrocytes and also found the LCN2 expression was significantly enhanced in both MPP^+^ and α‐Syn PFF‐treated astrocytes (Figure 1J,K). Collectively, the above findings suggest that LCN2 is associated with pathologic α‐Syn accumulation and is potentially involved in PD progression.

### 2.2 Astrocytic LCN2 Overexpression Aggravates PD‐Like Pathology and α‐Syn Accumulation in Mice

**Figure 2:**
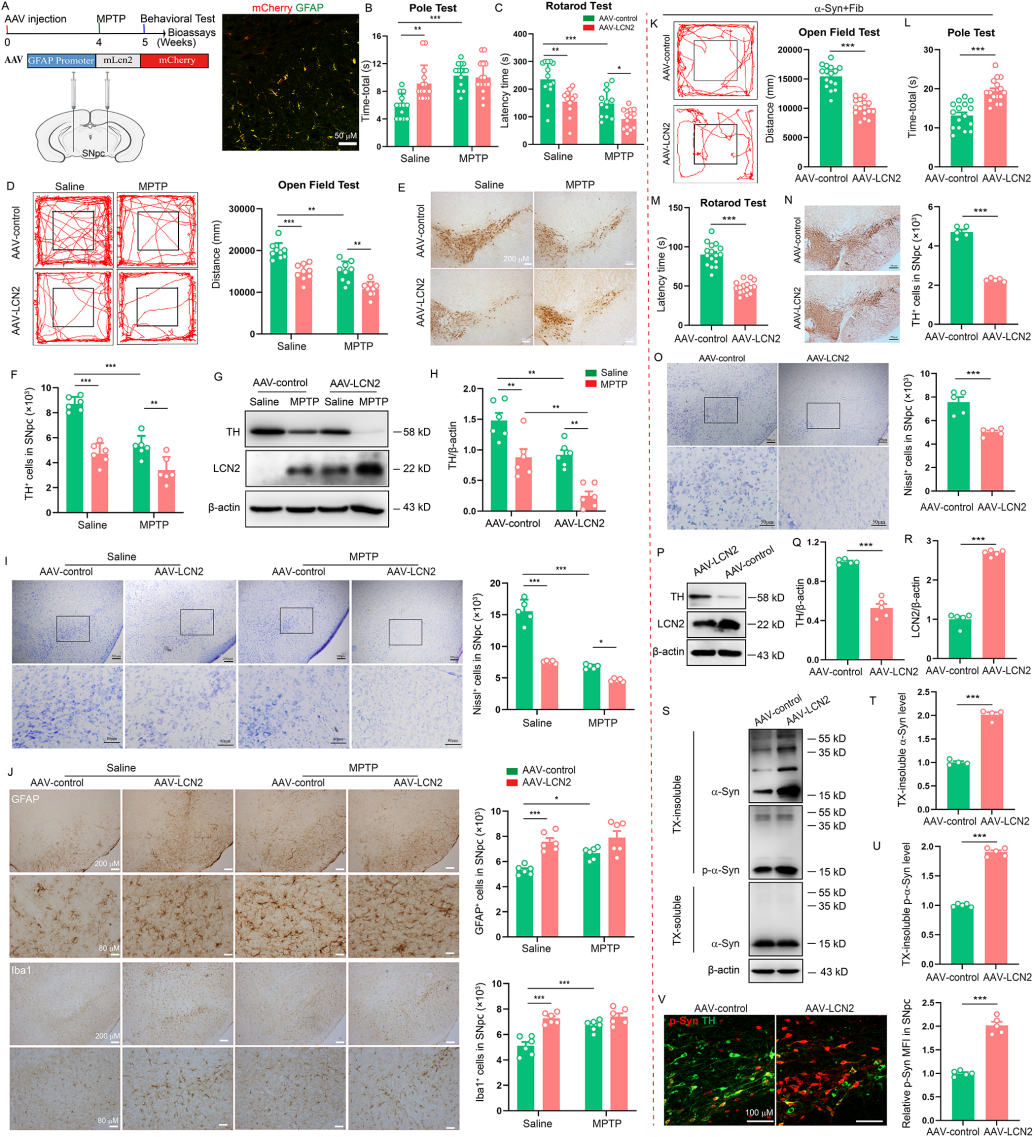
Astrocytic LCN2 overexpression aggravates PD‐like pathology and α‐Syn accumulation in mice. A) Diagram of the experimental design. IHC results confirm that AAV‐Lcn2 (mCherry) is expressed, mainly in GFAP^+^ astrocytes (green). B) The time taken to descend a pole (Time‐total) was recorded in pole test in MPTP model (n = 14 animals for each group). C) Time on the rod was measured by the rotarod test in MPTP model (n = 12 animals for each group). D) Movement distance within 5 min was recorded in open field test in MPTP model (n = 9 animals for each group). E,F) Microphotographs and stereological counts of TH‐positive neurons in the SNpc in MPTP model (n = 6 animals for each group). G,H) Representative immunoblots and quantification of relative expression of TH in the SNpc in MPTP model (n = 6 animals for each group). I) Immunohistochemical staining and quantification of Nissl‐positive cells in the SNpc in MPTP model. J) Microphotographs and quantification of GFAP‐positive astrocytes and Iba1‐positive microglia in the SNpc in MPTP model (n = 6 animals for each group). K) Movement distance within 5 min was recorded in AAV‐control or AAV‐LCN2‐injected mice in α‐Syn PFF model (n = 16 animals for each group). L) The time taken to descend a pole (Time‐total) was recorded in pole test in α‐Syn PFF model (n = 16 animals for each group). M) Time on the rod was measured by the rotarod test in α‐Syn PFF model (n = 16 animals for each group). N) Immunohistochemical staining and quantification of TH‐positive cells in the SNpc in α‐Syn PFF model (n = 5 animals for each group). O) Immunohistochemical staining and quantification of Nissl‐positive cells in the SNpc in α‐Syn PFF model (n = 5 animals for each group). P–R) Representative immunoblots (P) and quantification of relative expression of TH (Q) and LCN2 (R) in the SNpc in α‐Syn PFF model (n = 5 animals for each group). S–U) Representative immunoblots and quantification of relative expression of TX‐insoluble and TX‐soluble α‐Syn and p‐α‐Syn in the SNpc in α‐Syn PFF model (n = 4‐5 animals for each group). V) Representative double immunostaining for p‐Syn (red) and TH (green) in the SNpc in α‐Syn PFF model (n = 5 animals for each group). The data shown are the mean ± SEM. Two‐way ANOVA with Tukey's post‐hoc test was used (B–D, F, H–J) and Unpaired t test was used (K‐O, Q‐R, T‐V). ^*^p < 0.05, ^**^p < 0.01, ^***^p < 0.001.

To explore the role of astrocytic LCN2 in PD, we first injected Adeno‑associated virus (AAV) carrying GFAP promoter‐Lcn2 to the SNpc region to specifically enforce the LCN2 expression in astrocytes of MPTP model (**Figure** **2**A; Figure S3, Supporting Information). We found that astrocytic LCN2‐overexpressed mice showed significant behavior defects, including longer time in the pole test, shorter time in the rotarod test and fewer distance in open field test (Figure 2B–D). Furthermore, overexpression of LCN2 in astrocytes markedly aggravated DA neuron loss in the SNpc, as defined by TH immunostaining, Nissl staining, and western blot (WB) analysis (Figure 2E–I). In addition, astrocytic LCN2 overexpression significantly promoted the activation of microglia and astrocytes and increased iron accumulation in the SNpc (Figure 2J; Figure S4, Supporting Information). Meanwhile, mass spectrometry (MS) was performed, and KEGG pathway analysis showed that LCN2 was mainly associated with pathological protein aggregation and accumulation (Figure S5A, Supporting Information). Since α‐Syn is the main pathological marker of PD, we tested whether astrocytic LCN2 would affect α‐Syn accumulation in α‐Syn PFF model. As expected, we observed a significant increase in pathological p‐α‐Syn staining and insoluble α‐Syn and p‐α‐Syn in astrocytic LCN2 infected mice compared to control mice (Figure S5B,C, Supporting Information). We further confirmed the role of astrocytic LCN2 in α‐Syn PFF‐induced PD model. We found that astrocytic LCN2‐overexpressed mice showed significant behavior defects in α‐Syn PFF‐induced PD model, including longer time in the pole test, shorter time in the rotarod test and fewer distance in open field test, compared to those in control mice (Figure 2K–M). Furthermore, overexpression of LCN2 in astrocytes markedly aggravated DA neuron loss in the SNpc (Figure 2N–R). In addition, WB analysis for soluble/insoluble fractions showed that astrocytic LCN2 overexpression significantly increased the levels of insoluble α‐Syn and p‐α‐Syn in the SNpc (Figure 2S–U). Meanwhile, we also observed significant increase in p‐α‐Syn staining in astrocytic LCN2‐overexpressed mice (Figure 2V). These results indicate that LCN2 is important modulator for the progression of PD and α‐Syn accumulation.

### 2.3 Astrocytic LCN2 Ablation Alleviates α‐Syn Pathology in Mice

**Figure 3:**
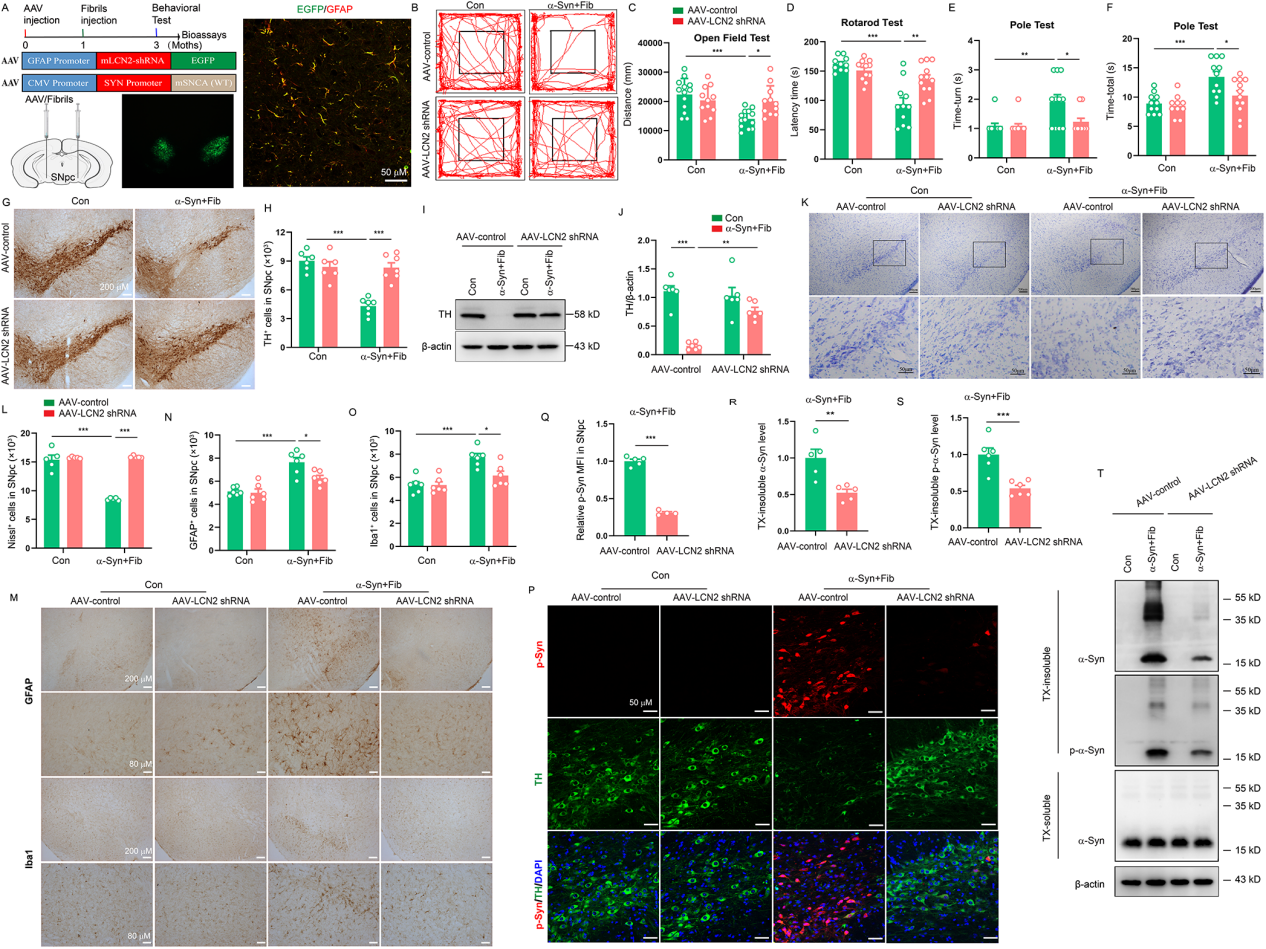
Astrocytic LCN2 ablation alleviates α‐Syn pathology. A) Diagram of the experimental design. IHC results confirm that AAV‐LCN2 shRNA (EGFP) is expressed, mainly in GFAP^+^ astrocytes (red) in α‐Syn PFF model. B,C) Movement distance within 5 min was recorded in open field test (n = 11‐13 animals for each group). D) Time on the rod was measured by the rotarod test (n = 11‐12 animals for each group). E‐F) the time taken to turn around (Time‐turn) and descend a pole (Time‐total) was recorded in pole test (n = 10‐12 animals for each group). G‐H) Microphotographs and stereological counts of TH‐positive neurons in the SNpc (n = 6‐7 animals for each group). I,J) Representative immunoblots and quantification of relative expression of TH in the SNpc (n = 6 animals for each group). K,L) Immunohistochemical staining and quantification of Nissl‐positive cells in the SNpc. M) Microphotographs of GFAP‐positive astrocytes and Iba1‐positive microglia in the SNpc. N,O) Stereological counts of GFAP‐positive astrocytes (N) and Iba1‐positive microglia (O) in the SNpc (n = 6 animals for each group). P) Representative double immunostaining for p‐Syn (red) and TH (green) in the SNpc. Q) Quantification of p‐Syn fluorescence intensity in P (n = 4‐5 animals for each group). DAPI stains nucleus (blue). R–T) Representative immunoblots and quantification of relative expression of TX‐insoluble and TX‐soluble α‐Syn and p‐α‐Syn in the SNpc (n = 5‐6 animals for each group). The data shown are the mean ± SEM. Two‐way ANOVA with Tukey's post‐hoc test was used (C‐F, H, J, L, N‐O) and Unpaired t test was used (Q‐S). ^*^p < 0.05, ^**^p < 0.01, ^***^p < 0.001.

To further explore the roles of astrocytic LCN2 in α‐Syn PFF‐induced neurodegeneration, we then stereotaxically injected AAV carrying GFAP‐promoter‐Lcn2‐shRNA‐EGFP to mouse SNpc regions to specifically down‐regulate astrocytic LCN2 expression and prepared α‐Syn PFF model (**Figure** **3**A; Figure S6, Supporting Information). We found that astrocytic LCN2 deletion significantly improved the behavior impairment of α‐Syn PFF‐injected mice, including shorter time in the pole test, better performance in the rotarod test and longer distance in the open field test (Figure 3B–F). Meanwhile, we also observed that astrocytes‐specific LCN2 deletion obviously reversed the loss of DA neurons in α‐Syn PFF‐injected mice, as defined by TH immunostaining, Nissl staining, and WB analysis (Figure 3G–L). In addition, astrocyte‐specific deletion of LCN2 notably inhibited the activation of microglia and astrocytes in SNpc of α‐Syn PFF‐injected mice, detectable by immunostaining (Figure 3M–O). Importantly, we observed substantial p‐α‐Syn staining in α‐Syn PFF model, but p‐α‐Syn staining is reduced by greater than 60% in astrocytic LCN2‐deficient mice (Figure 3P,Q). Immunoblot analysis also demonstrated a significant reduction in insoluble fractions of total α‐Syn and p‐α‐Syn in astrocytic LCN2 deletion mice (Figure 3R–T). Together, these findings demonstrate that astrocytic LCN2 ablation alleviates neurodegeneration through inhibition of α‐Syn accumulation.

### 2.4 LCN2 Limits α‐Syn PFF Uptake by Astrocytes in An Autocrine Manner

**Figure 4:**
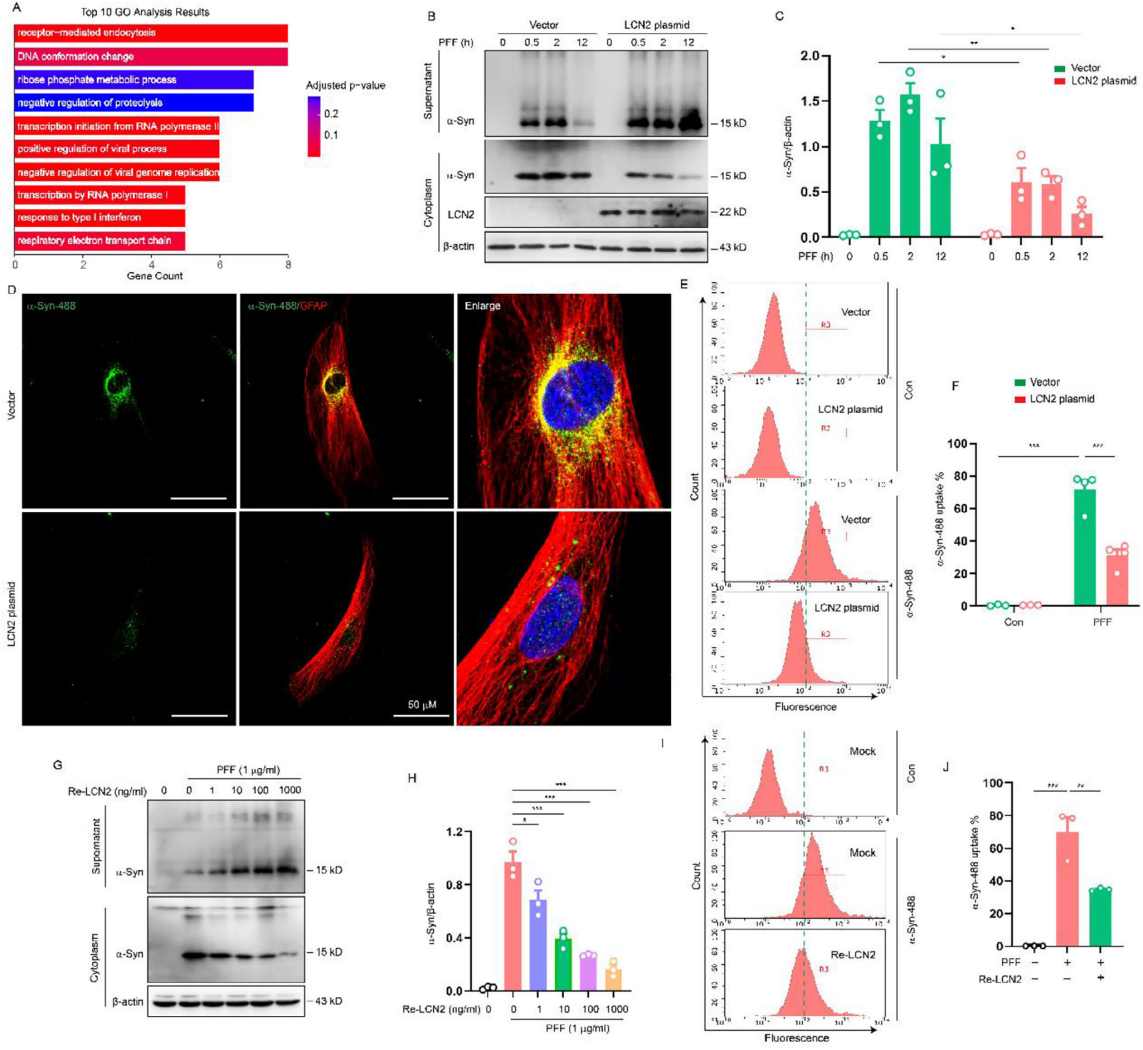
LCN2 limits α‐Syn PFF uptake by astrocytes in an autocrine manner. A) Top 10 GO enrichment analysis of biological processes for LCN2 by MS. B) Astrocytes were transfected with empty vector (Vector) or LCN2 plasmids for 48 h and were then stimulated with α‐Syn PFF (PFF, 1 µg mL^−1^) for 0.5, 2 or 12 h. Western blot analysis of α‐Syn in the supernatants and in the lysates (cytoplasm) of astrocytes. C) Quantification of relative level of α‐Syn in the lysates of astrocytes in B (Three independent experiments). D) Astrocytes were transfected with empty vector (Vector) or LCN2 plasmids for 48 h and then stimulated with Atto 488‐labeled α‐Syn PFF (1 µg mL^−1^) for 2 h. Representative image analysis of the endocytosis of α‐Syn‐488 PFF by astrocytes. E‐F) α‐Syn 488 PFF uptake in vector or LCN2‐expressed astrocytes was measured by flow cytometry (1 µg mL^−1^, 2 h of treatment, three or four independent experiments). G) Astrocytes were pretreated recombinant LCN2 protein (Re‐LCN2, 1, 10, 100, 1000 ng mL^−1^) for 30 min and were then stimulated with α‐Syn PFF (1 µg mL^−1^) for 2 h. Western blot analysis of α‐Syn in the supernatants and in the lysates (cytoplasm) of astrocytes. H) Quantification of relative level of α‐Syn in the lysates of astrocytes in G (Three independent experiments). I,J) α‐Syn 488 PFF uptake in mock or recombinant LCN2 protein (Re‐LCN2, 100 ng mL^−1^)‐pretreated astrocytes measured by flow cytometry (1 µg mL^−1^, 2 h of treatment, three independent experiments). The data shown are the mean ± SEM. Two‐way ANOVA with Tukey's post‐hoc test was used (C, F) and One‐way ANOVA with Tukey's post‐hoc test was used (H, J). ^*^p < 0.05, ^**^p < 0.01, ^***^p < 0.001.

To elucidate the underlying mechanisms, we applied MS and then performed GO analyses of biological processes, which revealed enrichment of receptor‐mediated endocytosis (**Figure** **4**A). Having demonstrated that astrocytic LCN2 is important modulator for the α‐Syn accumulation, we asked whether LCN2 would regulate α‐Syn PFF uptake/endocytosis by astrocyte. To test this, we re‐expressed LCN2 in astrocytes by transfecting the LCN2 gene and then treated the astrocytes with α‐Syn PFF, characterized by transmission electron microscopy (Figure S7, Supporting Information), for 0.5, 2, and 12 h at a concentration of 1 µg mL^−1^. We quantified the total intracellular α‐Syn in astrocytes by immunoblotting analysis and found that astrocytic overexpression of LCN2 significantly reduced α‐Syn uptake by astrocytes (Figure 4B,C). To further confirm the effects of LCN2 on α‐Syn PFF uptake, we treated the astrocytes with fluorescently labeled α‐Syn PFF for 2 h and measured the cellular α‐Syn signal by flow cytometry and confocal microscopy. We found that the enforced expression of LCN2 significantly reduced the cellular uptake of α‐Syn (more than 55% reduction) in the astrocytes (Figure 4D–F). Next, we tested whether the LCN2 could inhibit the α‐Syn uptake by astrocytes in an autocrine manner as a secreting protein. We treated the astrocytes with α‐Syn PFF (1 µg mL^−1^) for 2 h together with different concentrations of recombinant LCN2 protein ranging from 1 to 1000 ng mL^−1^. We found that recombinant LCN2 also strongly suppressed the astrocytic uptake of α‐Syn PFF in a concentration‐dependent manner, assessed by immunoblotting analysis (Figure 4G,H) and flow cytometry (Figure 4I,J). Overall, these results show astrocytic LCN2 limits α‐Syn PFF uptake by astrocytes in an autocrine manner.

### 2.5 LCN2 Receptor (24p3R) is An Astrocytic Receptor for α‐Syn PFF Uptake

**Figure 5:**
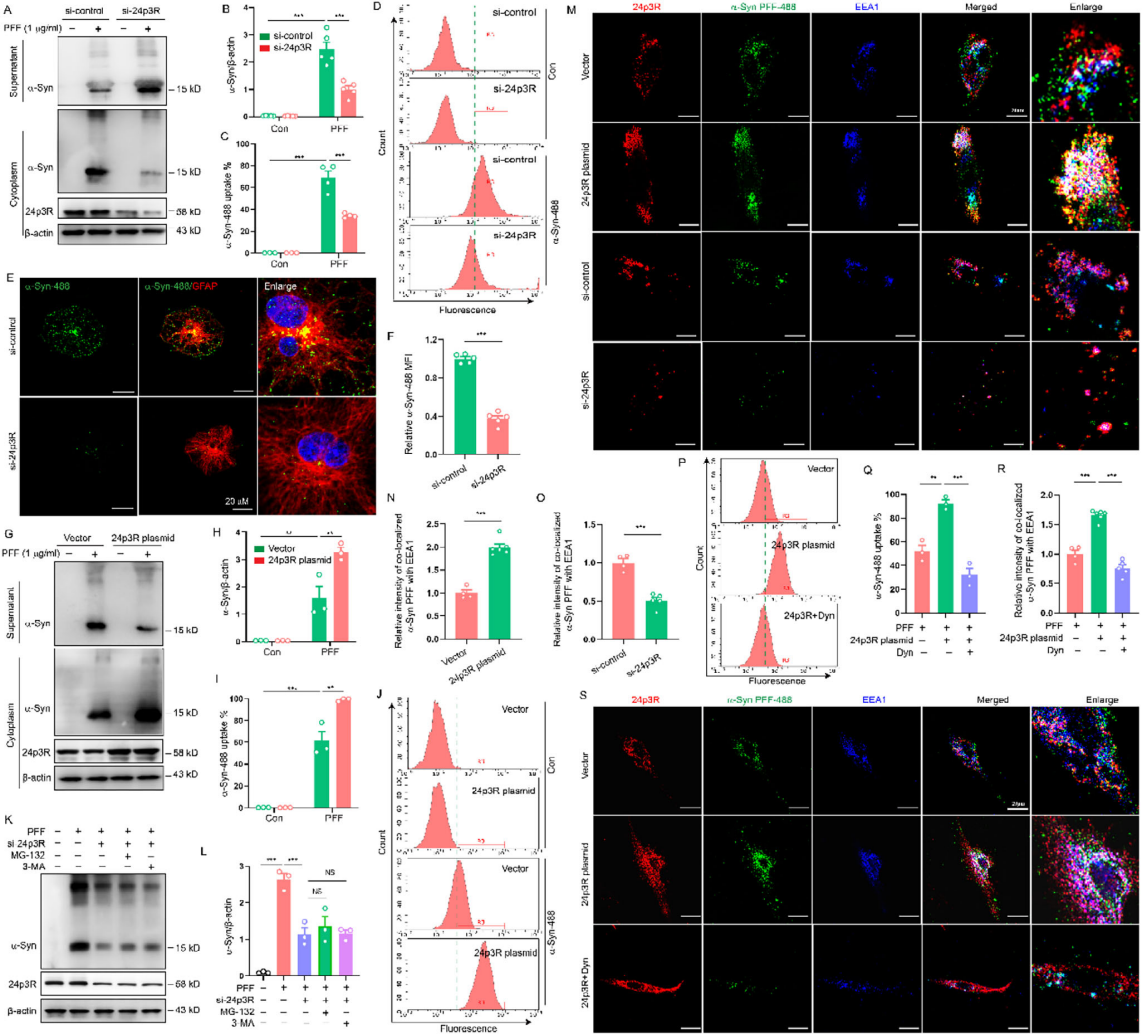
LCN2 receptor (24p3R) is an astrocytic receptor for α‐syn PFF uptake. A) Astrocytes were transfected with control siRNA (si‐control) or 24p3R siRNA (si‐24p3R) for 48 h and then treated with α‐Syn PFF (PFF, 1 µg mL^−1^) for 2 h. Western blot analysis of α‐Syn in the supernatants and in the lysates (cytoplasm) of astrocytes. B) Quantification of relative level of α‐Syn in the lysates of astrocytes in A (Five independent experiments). C,D) Astrocytes were transfected with control siRNA (si‐control) or 24p3R siRNA (si‐24p3R) for 48 h and were then stimulated with Atto 488‐labeled α‐Syn PFF (1 µg mL^−1^) for 2 h. α‐Syn 488 PFF uptake was measured by flow cytometry (1 µg mL^−1^, 2 h of treatment, three or four independent experiments). E,F) Representative image analysis of the endocytosis of α‐Syn‐488 PFF in control siRNA (si‐control) or 24p3R siRNA (si‐24p3R) transfected astrocytes (Five independent experiments). G) Astrocytes were transfected with empty vector (Vector) or 24p3R plasmids for 48 h and were then stimulated with α‐Syn PFF (1 µg mL^−1^) for 2 h. Western blot analysis of α‐Syn in the supernatants and in the lysates (cytoplasm) of astrocytes. H) Quantification of relative level of α‐Syn in the lysates of astrocytes in G (Three independent experiments). I,J) α‐Syn 488 PFF uptake in vector or 24p3R‐expressed astrocytes was measured by flow cytometry (1 µg mL^−1^, 2 h of treatment, three independent experiments). K,L) 24p3R siRNA‐transfected astrocytes were pretreated with MG‐132 (5 µM) or 3‐MA (10 mM) for 30 min and then treated with α‐Syn PFF (1 µg mL^−1^). Western blot analysis of α‐Syn in the lysates of astrocytes (Three independent experiments). M) Astrocytes were transfected with 24p3R plasmids or 24p3R siRNA (si‐24p3R) for 48 h and were then stimulated with Atto 488‐labeled α‐Syn PFF (1 µg mL^−1^) for 2 h. Internalized α‐syn‐488 PFF (green), 24p3R (red) co‐localized with EAA1 (blue) was assessed by confocal microscopy. N,O) Quantification of co‐localized α‐Syn 488 PFF with EAA1 in M (Three independent experiments). P,Q) 24p3R‐overexpressed astrocytes were pretreated with Dynasore (Dyn, 80 µM) for 30 min and then treated with Atto 488‐labeled α‐Syn PFF (1 µg mL^−1^) for 2 h. α‐Syn 488 PFF uptake in astrocytes was measured by flow cytometry (three independent experiments). R,S) Image and quantification of internalized α‐syn‐488 PFF (green), 24p3R (red) co‐localized with EAA1 (blue) in 24p3R‐overexpressed astrocytes treated with Dynasore (Dyn, 80 µM). The data shown are the mean ± SEM. Two‐way ANOVA with Tukey's post‐hoc test was used (B‐C, H‐I, L, Q‐R) and Unpaired t test was used (F, N‐O). ^**^p < 0.01, ^***^p < 0.001, NS: no significant.

Our data indicated that LCN2 modulated the α‐Syn uptake by astrocytes as a secreting protein. We supposed that LCN2 limited astrocytic α‐Syn uptake by binding to receptors. We first checked the expression of six putative LCN2 receptors, namely 24p3R, low density lipoprotein‐related protein 2 (LRP2), LRP6, melanocortin 4 receptor (MC4R), MC1R, and MC3R in astrocytes. We found that 24p3R was mainly expressed in astrocytes and significantly reduced after α‐Syn PFF treatment for 24 h and in PD mouse model, demonstrating that 24p3R is the primary LCN2 receptor in astrocytes (Figure S8, Supporting Information). To investigate whether 24p3R is involved in α‐Syn PFF uptake by astrocytes, we knocked down 24p3R in astrocytes using siRNA‐mediated gene silencing (Figure S9, Supporting Information). WB analysis showed that α‐Syn PFF uptake was significantly reduced in 24p3R siRNA astrocytes compared to control siRNA astrocytes (**Figure** **5**A,B). In addition, 24p3R deficiency markedly decreased the cellular uptake of α‐Syn in astrocytes, detectable by immunostaining and flow cytometry analysis (Figure 5C–F). Conversely, overexpression of 24p3R significantly enhanced the uptake of α‐Syn PFF by astrocytes (Figure 5G–J). To further determine that 24p3R affects α‐Syn PFF via endocytosis or degradation, we quantified the total intracellular α‐Syn in 24p3R siRNA astrocytes after blocking degradation with autophagy inhibitor 3‐MA or proteasome inhibitor MG‐132. There was a significant decrease in α‐Syn uptake by 24p3R‐deficent astrocytes compared to control cells, but this decrease was not reversed after treatment with 3‐MA or MG‐132 (Figure 5K,L), suggesting that 24p3R modulates α‐Syn PFF may be not through degradation. EEA1 is an early endosomal marker and helps to mediate internalization.^[^ ^12^ ^]^ As such, we sought to confirm the internalization of α‐Syn PFF into endosomes by measuring the intensity of co‐localized α‐Syn PFF with EEA1. We found that 24p3R, EEA1 and α‐Syn PFF were co‐localized in astrocytes (Figure 5M). Overexpression of 24p3R in astrocytes increased the intensity of α‐Syn PFF co‐localizing with EEA1 (Figure 5N), while knockdown of 24p3R in astrocytes reduced the intensity of α‐Syn PFF co‐localizing with EEA1 (Figure 5O). To further dissect the mechanism underlying 24p3R‐meidated α‐Syn PFF endocytosis, we used a chemical blocker of endocytosis Dynasore. We showed that the α‐Syn PFF uptake was significantly enhanced in 24p3R‐overexpressed astrocytes and this enhancement was abrogated after Dynasore treatment, assessed by confocal microscopy and flow cytometry analysis (Figure 5P–S), demonstrating that 24p3R modulates α‐Syn PFF uptake via 24p3R‐mediated endocytosis in astrocytes. Together, these results indicate that 24p3R is an astrocytic receptor for α‐Syn PFF uptake.

### 2.6 24p3R Specifically Binds to α‐Syn PFF in Astrocytes

**Figure 6:**
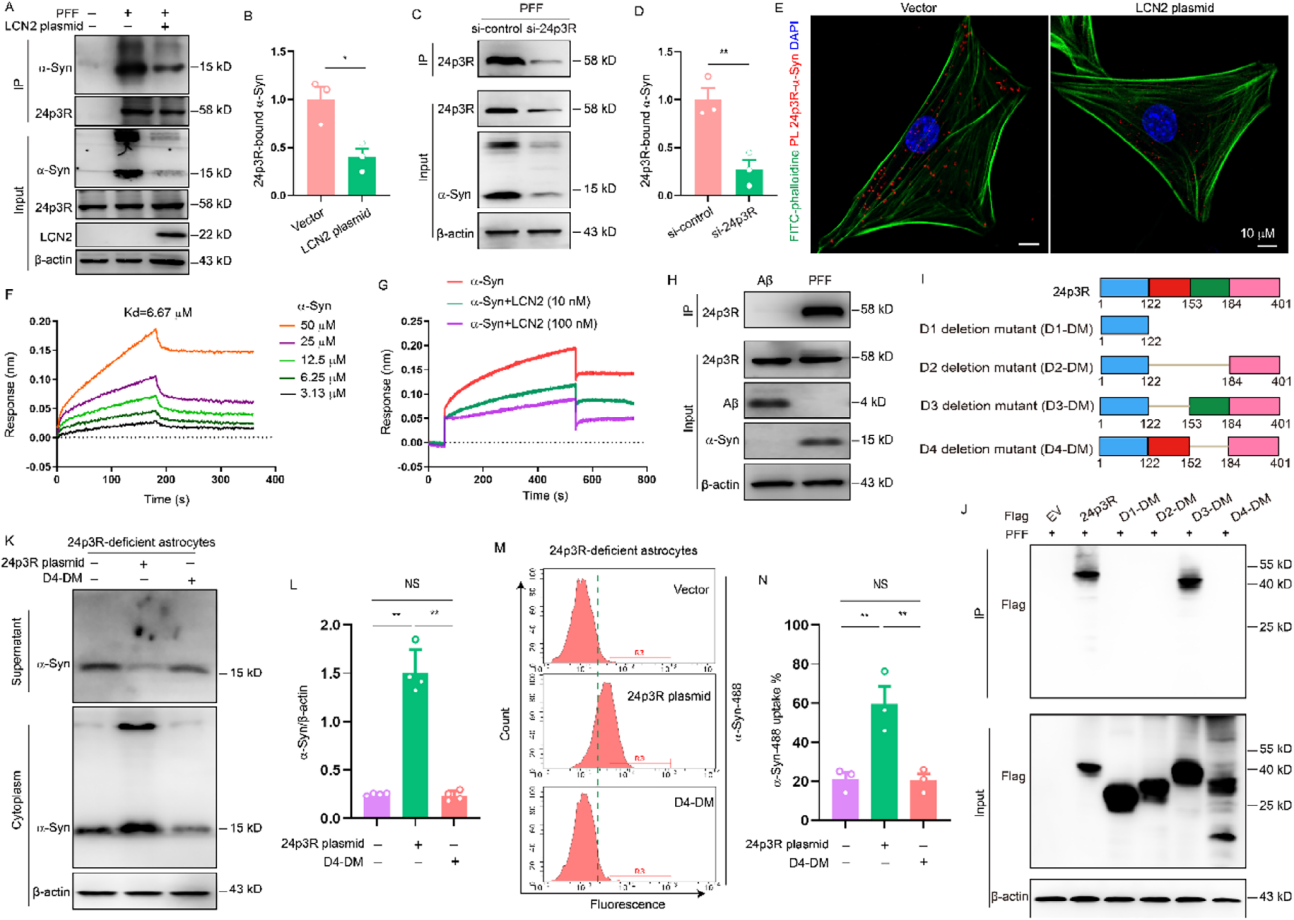
24p3R specifically binds to α‐Syn PFF in astrocytes. A) Astrocytes were transfected with empty vector (Vector) or LCN2 plasmids for 48 h and were then stimulated with α‐Syn PFF (PFF, 1 µg mL^−1^) for 2 h. Immunoprecipitation and immunoblot analysis of the interaction of 24p3R with α‐Syn in astrocytes. B) Quantification of 24p3R‐α‐Syn interaction in vector or LCN2 plasmids transfected astrocytes (Three independent experiments). C,D) Immunoprecipitation and immunoblot analysis of the interaction of 24p3R with α‐Syn in control siRNA (si‐control) or 24p3R siRNA (si‐24p3R) transfected astrocytes (Three independent experiments). E) 24p3R and α‐Syn proximity ligation signals in vector or LCN2 plasmids transfected astrocytes. F) SPR assay to evaluate the affinity between α‐Syn and purified mouse 24p3R protein. G) SPR assay to evaluate the affinity between α‐Syn and purified mouse 24p3R protein in absence or presence of LCN2. H) Astrocytes were treated with α‐Syn PFF (PFF, 1 µg mL^−1^) or amyloid‐beta (Aβ1‐42 fibrils, 1 µg mL^−1^) for 2 h. Immunoprecipitation and immunoblot analysis of the interaction of 24p3R with α‐Syn or Aβ in astrocytes. I) Schematic diagram of mouse 24p3R domains and deletions mutants. J) HEK293T cells were transfected with empty vector (EV) or expression plasmids directing the expression of each of the indicated 24p3R deletion mutants. Transfected cells were assessed for binding of α‐Syn. K) 24p3R deficient astrocytes were transfected with 24p3R plasmids or 24p3R D4 deletion mutant (D4‐DM) for 48 h and then treated with α‐Syn PFF (1 µg mL^−1^) for 2 h. Western blot analysis of α‐Syn in the supernatants and in the lysates (cytoplasm) of astrocytes. L) Quantification of relative level of α‐Syn in the lysates of astrocytes in K (Four independent experiments). M,N) α‐Syn 488 PFF uptake in 24p3R deficient astrocytes transfected with 24p3R plasmids or 24p3R D4 deletion mutants (D4‐DM) was measured by flow cytometry (1 µg mL^−1^, 2 h of treatment, three independent experiments). The data shown are the mean ± SEM. Unpaired t test was used (B, D) and two‐way ANOVA with Tukey's post‐hoc test was used (L, N). ^*^p < 0.05, ^**^p < 0.01.

To test whether LCN2/24p3R was able to bind α‐Syn PFF, we first performed the physical interaction between the LCN2/24p3R and α‐Syn PFF by immunoprecipitation. We found that LCN2 failed to interact with α‐Syn (Figure S10, Supporting Information). Interestingly, 24p3R could bind α‐Syn in astrocytes and this binding was significantly reduced by LCN2 overexpression or 24p3R knockdown (**Figure** **6**A–D). Then, the interaction of 24p3R and α‐Syn PFF was further confirmed by PLA, a highly sensitive and specific method of assessing interactions between two proteins (Figure 6E). Lastly, SPR was used to measure the binding of α‐Syn PFF to recombinant 24p3R. Interestingly, we found a direct protein‐protein interaction between 24p3R and α‐Syn PFF and this binding was significantly reduced in the presence of LCN2 (Figure 6F,G). In contrast, 24p3R failed to bind to β‐amyloid, indicating that 24p3R specifically binds to α‐Syn PFF (Figure 6H). To determine the α‐Syn PFF binding domain, we deleted D1 domain (123‐401 aa), D2 domain (123‐183 aa), D3 domain (123‐152 aa) or D4 domain (153‐183 aa) of 24p3R and performed the co‐immunoprecipitation with overexpression of 24p3R deletion mutants (Figure 6I). The results showed that α‐Syn PFF preferentially binds to the D4 domain of 24p3R (Figure 6J), suggesting that 24p3R residues 153–183 in the D4 domain are responsible for the binding between 24p3R and α‐Syn PFF.

We next sought to determine whether 24p3R regulates α‐Syn PFF uptake through the binding to α‐Syn PFF. We re‐expressed 24p3R full length or D4 domain deletion mutant in 24p3R‐deficient astrocytes and then measured the internalization of α‐Syn PFF by astrocytes. We found that transfection of 24p3R full length effectively restored the uptake of α‐Syn PFF in 24p3R‐deficient astrocytes, but overexpression of D4 domain deletion mutant failed to restore the uptake of α‐Syn PFF by astrocytes (Figure 6K–N), indicating that 153–183 residues of 24p3R are critical for 24p3R‐mediated α‐Syn PFF uptake.

### 2.7 Astrocytic 24p3R Deletion Promotes α‐Syn Spread and Toxicity in α‐Syn PFF Model

**Figure 7:**
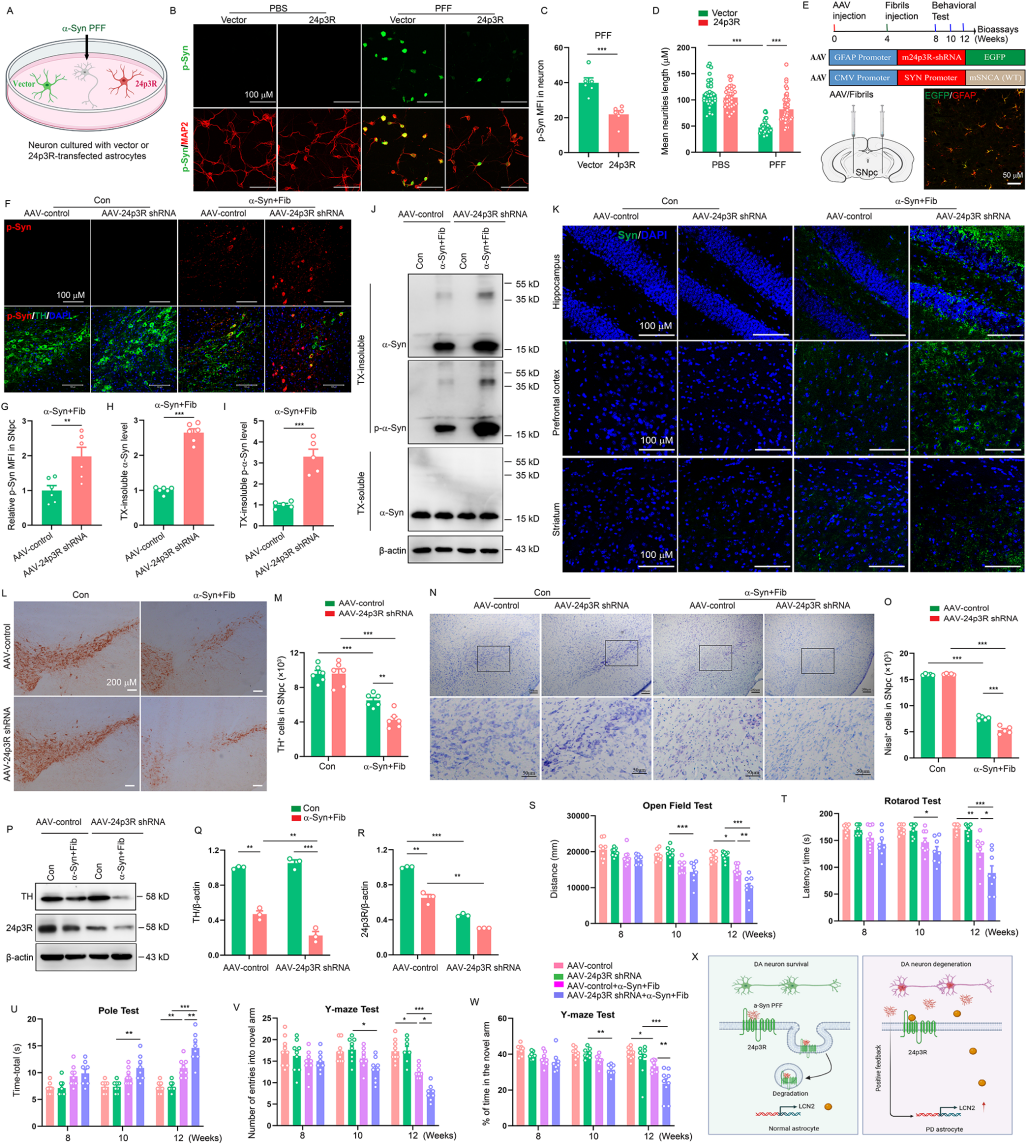
Astrocytic 24p3R deletion promotes α‐Syn spread and toxicity in α‐Syn PFF model. A) Schematic representation of the chambers in which neurons were co‐cultured with vector or 24p3R‐overexpressed astrocytes. α‐Syn PFF (1 µg mL^−1^) was added to chambers for 10 days. B) Representative double‐immunostaining for p‐α‐Syn (green) and MAP2 (red) when neurons were co‐cultured with vector or 24p3R‐overexpressed astrocytes after α‐Syn PFF (1 µg mL^−1^) treatment for10 days. C,D) Quantification of p‐α‐Syn in neuron and mean total neurites length in B (Six independent experiments). E) Diagram of the experimental design. IHC results confirm that AAV‐24p3R shRNA (EGFP) is expressed, mainly in GFAP^+^ astrocytes (red) in α‐Syn PFF model. F) Representative double immunostaining for p‐Syn (red) and TH (green) in the SNpc. G) Quantification of p‐Syn fluorescence intensity in F (n = 6 animals for each group). DAPI stains nucleus (blue). H–J) Representative immunoblots and quantification of relative expression of TX‐insoluble and TX‐soluble α‐Syn and p‐α‐Syn in the SNpc (n = 5‐6 animals for each group). K) Representative images showing α‐Syn signals in the hippocampus, prefrontal cortex and striatum region from AAV‐control and AAV‐24p3R shRNA mice. L,M) Microphotographs and stereological counts of TH‐positive neurons in the SNpc (n = 6 animals for each group). N,O) Immunohistochemical staining and quantification of Nissl‐positive cells in the SNpc (n = 5 animals for each group). P‐R) Representative immunoblots (P) and quantification of relative expression of TH (Q) and LCN2 (R) in the SNpc (n = 3 animals for each group). S) Movement distance within 5 min was recorded in open field test (n = 8 animals for each group). T) Time on the rod was measured by the rotarod test (n = 8‐10 animals for each group). U) The time taken to descend a pole (Time‐total) was recorded in pole test (n = 8 animals for each group). V,W) The percentage of time spent in the novel arm and the number of entries into novel arm were recorded in Y maze test (n = 8‐10 animals for each group). X) Proposed model for how LCN2 impedes the α‐Syn uptake by astrocytes in PD. The data shown are the mean ± SEM. Unpaired t test was used (C, G‐I) and two‐way ANOVA with Tukey's post‐hoc test was used (D, M, O, Q‐W). ^*^p < 0.05, ^**^p < 0.01, ^***^p < 0.001.

Next, we examined whether 24p3R‐mediated a‐Syn uptake by astrocytes affects a‐Syn levels and toxicity in neurons. Using coculturing primary neurons and astrocytes, we re‐expressed 24p3R in astrocytes and added α‐Syn PFF to cultures. Ten days after α‐Syn PFF treatment, the levels of p‐α‐syn in neurons were markedly increased when neurons were cocultured co‐cultured with vector‐expressed astrocytes, while the levels of p‐α‐syn in neurons were significantly reduced when neurons were cocultured co‐cultured with 24p3R‐expressed astrocytes (**Figure** **7**A–C). In addition, astrocytic 24p3R overexpression prevented the α‐Syn PFF‐induced the neuron injury (Figure 7D). Thus, 24p3R‐mediated a‐Syn uptake by astrocyte protects neurons from a‐Syn accumulation and toxicity.

Subsequently, to teste whether astrocytic 24p3R was also critical for the spread of α‐Syn in vivo, we stereotaxically injected AAV carrying GFAP‐promoter‐24p3R‐shRNA‐EGFP to the SNpc regions to specifically down‐regulate mouse astrocytic 24p3R expression in α‐Syn PFF model (Figure 7E,R). As expected, p‐α‐Syn staining in the SNpc was significantly increased in astrocytic 24p3R deleted mice compared with control mice (Figure 7F,G). In addition, immunoblot analysis also demonstrated a significant increase in insoluble fractions of total α‐Syn and p‐α‐Syn in the SNpc of astrocytic 24p3R deletion mice (Figure 7H–J). Interestingly, in the hippocampus, cortex and striatum above SNpc, we also found α‐Syn signals in astrocytic 24p3R deleted mice (Figure 7K), indicating that astrocytic 24p3R deletion promotes the spread of α‐Syn in mouse brain. Accompanying the α‐Syn pathology, stereologic counting of the SNpc cells revealed significant loss of DA neurons and more glial cell activation in the astrocytic 24p3R‐deficient mice compared to controls (Figure 7L–O; Figure S11, Supporting Information). In addition, astrocytes‐specific 24p3R deletion obviously reduced the expression of TH protein in the SNpc, as defined by immunoblot analysis (Figure 7P,Q). More importantly, at 10 weeks post‐α‐Syn PFF injection, astrocytic 24p3R‐deficient mice exhibited significant impairment in motor function and cognition, whereas control mice showed no significant impairments. At 12 weeks post‐α‐Syn PFF injection, both astrocytic 24p3R‐deficient mice and control mice showed motor and cognitive impairment, but the impairment in 24p3R‐deficient mice was more severe than in control mice (Figure 7S–W). Therefore, astrocytic 24p3R is crucial for α‐Syn PFF‐induced neurodegeneration and the development of PD related motor and cognitive defects.

## 3 Discussion

The major finding of this paper is that 24p3R is an astrocytic receptor for α‐Syn uptake. The progressive accumulation and spread of insoluble aggregates of the a‐Syn is a hallmark of neurodegenerative disorders including PD.^[^ ^13^ ^]^ Strategies to prevent cell‐to‐cell spread of α‐Syn have been proposed as promising therapies to prevent neurodegeneration in synucleinopathies.^[^ ^14^ ^]^ Various molecular mechanisms related to the transmission of α‐Syn pathology have been reported, including the receptor‐mediated endocytosis.^[^ ^15^ ^]^ Many receptors facilitating the uptake of a‐Syn aggregates were identified across brain cell types.^[^ ^16^ ^]^ For instance, heparan sulfate proteoglycans,^[^ ^17^ ^]^ Α3‐subunit of the Na^+^/K^+^‐ATPase^[^ ^18^ ^]^ cellular prion protein^[^ ^19^ ^]^ and lymphocyte‐activation gene 3^[^ ^20^ ^]^ have been identified as mediators of α‐Syn PFF in internalization by neurons. MerTK and Toll‐like receptor 2 are essential receptors that mediate α‐Syn PFF uptake by microglia.^[^ ^21^ ^]^ However, the receptor that mediates α‐Syn PFF uptake by astrocytes remains unclear. In the present study, we identified 24p3R as an essential receptor that mediated α‐Syn PFF uptake by astrocytes. Using loss of function and gain of function, we showed that 24p3R‐deficiency significantly reduced α‐Syn PFF uptake by astrocytes, whereas 24p3R overexpression markedly increased α‐Syn PFF uptake, indicating that α‐Syn PFF internalization by astrocytes is dependent on 24p3R. Importantly, we also confirmed that mice lacking astrocytic 24p3R exhibited accelerated α‐Syn spread and aggravated α‐Syn PFF induced pathology and toxicity in α‐Syn PFF model. Our findings support the hypothesis that 24p3R is a key regulator for α‐Syn accumulation and spread, providing potential therapeutic insight targeting the relevant diseases with α‐Syn pathogenesis.

As a cell surface receptor, 24p3R is enriched in the brain.^[^ ^22^ ^]^ Our finding indicated that 24p3R was expressed predominantly in astrocytes, consistent with the observation that 24p3R mediated the uptake of α‐Syn PFF by astrocytes. To elucidate the molecular mechanism underlying 24p3R regulating α‐Syn PFF uptake, by using multiple techniques, we showed that 24p3R specifically bound to α‐Syn PFF, but not β‐amyloid. Moreover, we identified that 24p3R residues 153–183 are responsible for the binding to α‐Syn PFF and α‐Syn PFF uptake by astrocytes.

Accumulating evidence indicates that aggregated α‐Syn species released by degenerating neurons can actively contribute to the spreading of PD pathology.^[^ ^23^ ^]^ Therefore, the clearance of extracellular α‐Syn toxic forms is crucial to control the propagation and the progression of the disease.^[^ ^24^ ^]^ Astrocytes have been identified as a promising cell type in the clearance of extracellular α‐Syn.^[^ ^25^ ^]^ However, underlying molecular mechanisms responsible for clearance of extracellular α‐Syn by astrocytes is poorly understood. Here, we provided evidence that the LCN2 limited the clearance of α‐Syn PFF by astrocytes through competitively impeding the binding of 24p3R and α‐Syn PFF. It has been reported that LCN2 regulates inflammation,^[^ ^26^ ^]^ but its role in α‐Syn PFF uptake and clearance remains unclear. In the present study, we showed that the LCN2 was increased in astrocytes from both MPTP and α‐Syn PFF model. When LCN2 was secreted from astrocytes, it bound to its receptor 24p3R in astrocytes and this binding impeded the α‐Syn PFF binding to 24p3R. This in turn prevented 24p3R‐mediated α‐Syn PFF uptake and reduced the clearance of extracellular α‐Syn by astrocytes, which contributed to the spreading of PD pathology (Figure 7X). Therefore, LCN2 limited α‐Syn PFF uptake by astrocytes in an autocrine manner as a secreting protein. In addition to this, we also found that LCN2 overexpression in astrocytes alone induced DA neuron degeneration and glial cell activation, which was accompanied by an increase in iron levels, suggesting that iron dysregulation may be involved in LCN2‐mediated neurotoxicity in the absence of α‐Syn pathology. Although our study has provided compelling data showing the role of LCN2 in 24p3R‐mediated uptake of a‐Syn by astrocytes, we do not exclude the possibility that other pathways or other receptors also at play. Interestingly, we observed that LCN2 level was elevated in α‐Syn PFF‐treated astrocytes. As NF‐κB and YYI are potential transcriptional regulators of LCN2,^[^ ^1^, ^8^ ^]^ we speculate that α‐Syn PFFs may activate pro‐inflammatory pathways (e.g., TLR4/NF‐κB) or inhibit YY1 to upregulate LCN2, while 24p3R downregulation may reflect feedback inhibition due to elevated LCN2 in the α‐Syn PFF model. This increased LCN2 inhibited the clearance of extracellular α‐Syn by astrocytes and then promoted α‐Syn spreading and accumulation, thereby further upregulating LCN2. Ultimately, the LCN2/α‐Syn positive feedback loop exacerbated PD pathology. Moreover, we showed that interruption of this loop by deleting the Lcn2 gene in astrocytes promoted α‐Syn PFF uptake by astrocytes, which prevented α‐Syn spreading and accumulation in neuron and thereby alleviated PD pathology. Thus, our study illustrated the concept that inhibiting this vicious feedback loop represented a potential therapeutic strategy for treating the relevant diseases with α‐Syn pathogenesis, including PD.

Collectively, our findings define 24p3R as a key receptor for α‐Syn uptake in astrocytes. Moreover, we propose a LCN2/α‐Syn positive feedback loop that limits the uptake of α‐Syn by astrocytes, thereby exacerbating the spreading of α‐Syn pathology in α‐Syn PFF model. Thus, our study illustrates that disruption of the vicious positive feedback loop may be a potential therapeutic approach for the treatment of PD and related α‐synucleinopathies.

## 4 Experimental Section

### Experimental Animals

Mice (C57Bl/6 background) were maintained at the Animal Resource Centre of the Faculty of Medicine (Nanjing Medical University) and housed under standard laboratory conditions (22 ± 1 °C, 12 h light‐dark cycle, food and water ad libitum).

### Ethical Statement

All procedures concerning animal care and treatment were performed in accordance with the protocols approved by the Institutional Animal Care and Use Committee (IACUC‐2008067) of Nanjing Medical University.

### Preparation of AAV particles

AAV particles (AAV‐m‐Lcn2‐GFAP promoter‐MCS 3FLAG hGH PolyA, GOSV0334717) were purchased from Shanghai GeneChem Co. (Shanghai, China). pAAV‐GFaABC1D‐EGFP‐3×FLAG‐miR30‐shRNA (mSlc22a17)‐WPRE were purchased from OBiO Technology (Shanghai, China). rAAV‐GFaABC1D‐EGFP‐5′miR‐30a‐shRNA (mLcn2)‐3′‐miR30a‐WPREs and rAAV‐CMV‐SYN‐SNCA (WT)‐WPRE‐bGHpA were purchased from BrainVTA Technology (Wuhan, China).

### MPTP/p‐Induced PD Mouse Model

The MPTP protocol was performed as described previously.^[^ ^27^ ^]^ The randomly mice (male, 16 week) were injected subcutaneously with MPTP hydrochloride (25 mg kg^−1^, Sigma–Aldrich, St. Louis, MO, USA) followed by intraperitoneal injection of probenecid (250 mg kg^−1^, Sigma, St. Louis, MO, USA) at 1 h interval for 5 consecutive days and left for 3 days.

### α‐Syn PFF‐Induced PD Mouse Model

As previously described,^[^ ^28^ ^]^ 0.5 µL of AAV‐α‐Syn was delivered into the SN at a rate of 0.2 µl min^−1^ using the following coordinates: −3.0 mm A/P, ±1.2 mm M/L, and −4.5 mm D/V from bregma. After 1 month, α‐Syn PFF (Abcam, ab218819, 5 µg for each mouse) were briefly sonicated and stereotactically injected into the SN at a rate of 0.2 µl min^−1^ for 2 months.

### Stereotaxic Injection

The surgical procedure was carried out as previously described.^[^ ^1^ ^]^ Under anesthesia, the 1 µL of AAV was bilaterally delivered into the SN at a rate of 0.2 µl min^−1^ using the following coordinates: −3.0 mm A/P, ±1.2 mm M/L, and −4.5 mm D/V from bregma.

### Behavioral Analysis

Behavioral analyses were performed as described previously.^[^ ^29^ ^]^ For the pole test, the mice were placed head upward on the top of a vertical wooden rough‐surfaced pole (diameter 1 cm, height 50 cm). The total time until the mouse reached the floor with its four paws was recorded (T‐total). The time needed for the mouse to turn completely head downward was recorded (T‐turn). For the rotarod test, mice were accustomed to the apparatus before testing. The mice were then placed on the rod and tested at 20 rpm for 300 s. The latency time that each mouse stayed on the rod at rotarod speed was recorded. Locomotor activity of mice was detected in an activity monitor. The mouse was placed into activity monitor chambers (20 cm × 20 cm × 15 cm) for 30 min, and the activities were recorded at 5‐min intervals. For the Y‐Maze test, the maze is randomly defined as three arms, including the novel arm, starting arm and other arm. During the training time, the mice were placed in the starting arm to explore activities freely in the starting arm and other arm for 5 min when the novel arm was closed. During the test time, the novel arm was opened and then the mice to move freely throughout 3 arms for 5 min. The percentage of time traveled in the novel arm and the number of entries into novel arm were recorded and analyzed. The tester was blinded to all genotypes and treatment groups for each behavioral testing.

### Immunohistochemistry and Immunofluorescence

Brain tissue encompassing each midbrain was cut into 30‐µm slices using a freezing microtome (Leica M1950, Nussloch, Germany) as described previously.^[^ ^30^ ^]^ Brain slices were incubated with mouse antibody against TH (T1299, 1:3000, Sigma, USA), rabbit antibody against GFAP (MAB360, 1:1000, Millipore, USA), or rabbit antibody against Iba1 (17 198, 1:500, Cell Signaling Technology, USA) for the detection of TH‐positive neuron, astrocyte and microglia, respectively, overnight and then for 1 h with secondary antibodies. Immunoreactivity was visualized by incubation in substrate‐chromogen solution (DAB). Control staining was performed without primary antibodies. The total number of TH‐positive neurons, astrocyte and microglia in the SNpc were counted stereologically using the Optical Fractionator (Stereo Investigator 7, MBF bioscience, Williston, VT, USA).

For immunofluorescence staining, the slices or the astrocytes were incubated with anti‐GFAP (MAB360, 1:1000, Millipore, USA), anti‐TH (T1299, 1:3000, Sigma, USA), anti‐Iba1 (17 198, 1:500, Cell Signaling Technology, USA), anti‐LCN2 (AF1757, 1:200, R&D), anti‐24p3R (SAB3500306, 1:200, Sigma–Aldrich), anti‐EEA1 (AF8047, 1:200, R&D), anti‐MAP2 (sc‐74421, 1:1000, Santa Cruz Biotechnology), anti‐α‐Syn (610 786, 1:500, BD) or anti‐p‐α‐Syn (Ab51253, 1:400, Abcam) overnight at 4 °C and then incubated with Alexa Fluor 555‐conjugated antibody (Invitrogen, A21432; 1:1000) or Alexa Fluor 488‐conjugated antibody (Invitrogen, A21202; 1:1000) for 1 h at 20 °C. DAPI (P36931, Life Technologies) visualizes nuclei. Images were acquired by a confocal microscope (Axiovert LSM510, Carl Zeiss Co., Germany) and then processed by Image J.

### Nissl Staining

Nissl Staining Solution (C0117, Beyotime Biotechnology) was used to stain the Nissl body of the brain slices. A bluish‐purple color was observed to display the basic nervous structure of the brain. Images were captured by a fluorescence microscope, and ImageJ software was used to analyze stained cells.

### Perls Prussian Blue Staining

Brain slices underwent a thorough washing procedure with PBS prior to Perls Prussian Blue staining (G1428, Solarbio, China). Subsequent to completing the staining protocol as per the provided instructions, the brain slices underwent sequential dehydration in 70% ethanol for 30 s, 80% ethanol for 60 s, 90% ethanol for 60 s, and absolute ethanol for 2 min. Following this, the slices were cleared in xylene for 5 min. Following the application of neutral resin for mounting, the slices underwent observation and microscopic imaging.

### RNA Sequencing

Total messenger RNA (mRNA) was extracted from the SNpc tissues of control and MPTP‐treated mice. RNA libraries were sequenced on the Illumina sequencing platform by LC‐BIO Co., Ltd (HangZhou, China). Differentially expressed genes were defined as fold changes cutoff with |log2 ratio| ≥ 1 and p<0.05.

### Liquid Chromatograph—Tandem Mass Spectrometry (LC‐MS/ MS)

Astrocytes were lysed, centrifuged and the supernatants were incubated with anti‐LCN2 (AF1757, R&D) antibodies at 4 °C overnight and precipitated with protein A/G‐agarose beads. Immunocomplex of anti‐LCN2 antibodies was resolved in 1D‐PAGE gel followed by silver staining of the gel. Protein bands were excised and digested with trypsin. Peptides were extracted and analyzed using the automated LC‐MS/MS method according to procedure. LC‐MS/MS was performed and pathway was analyzed by Applied Protein Technology Corporation (APTBIO, Shanghai, China).

### Quantitative RT‐PCR (qPCR)

Total RNA was extracted from the SNpc tissue with Trizol reagent (Invitrogen, USA). Reverse transcription PCR was carried out using a TAKARA PrimeScript RT reagent kit and qPCR was performed in duplicate for each sample using a QuantiTect SYBR Green PCR kit (Qiagen, Germany) with an ABI 7300 Fast Real‐Time PCR System (Applied Biosystems, Foster City, CA, USA). GAPDH was used as an internal control for the real‐time PCR amplification. The sequences of primers used are as follows: Plin4 forward: GACCAGCAGTGAAGATGCCT, reverse: TCCTTCGTATTGGTGAGGACA. Ch25h forward: GTGCTGGACGTCCTGTATCC, reverse: AGCACGTCGAAGAAGGTCAG. Tox2 forward: TGATGGTGACAGTGCCTACGTG, reverse: GGGAGATTGGGAGGCGTTAT. Dbh forward: GGACCCCGAAGGGATTTTAGA, reverse: CCATCTCTCCTCGATCTGACA. Selp forward: CATCTGGTTCAGTGCTTTGATCT, reverse: ACCCGTGAGTTATTCCATGAGT. Mfap3 forward: CTTCTTACTGGTGGTCACTCTTG, reverse: CCGAGTAGGAACCTGCTGAGA. Lcn2 forward: AGCTTTACGATGTACAGCACCAT, reverse: GATACCTGTGCATATTTCCCAGA. Scgb1a1 forward: ATGAAGATCGCCATCACAATCAC, reverse: GGATGCCACATAACCAGACTCT. Zfp46 forward: CGGAAATGTTGTCTCACTGGA, reverse: ATCGTCACTAAACTCCTGTTTGG. Bpifa1 forward: TGCCTTTGGCTGTAAGCCC, reverse: AGAATTGCCTCCTCCAGACTTTA. Scgb3a2 forward: CCACTGCCCTTCTCATCAACC, reverse: TGTCGTCCAAAGGTACAGGTA. Ppp1r26 forward: CTCATCAGCACGCTTCAGAGG, reverse: TCGGTCCACGGAGTCATCA. Cxcl15 forward: TCGAGACCATTTACTGCAACAG, reverse: CATTGCCGGTGGAAATTCCTT. Sftpc forward: ATGGAGAGTCCACCGGATTAC, reverse: ACCACGATGAGAAGGCGTTTG. Sec14l3 forward: GCCATGCTCCGCAAGTACAT, reverse: CCATCACGGTCATAGCCACAC. Erbb4 forward: AGCCTGGTGAGATGGTGTTTG, reverse: GTGCTATGGACCCTACGTTAGT. Mmp8 forward: GGGCTCTGAGTGGCTATGAT, reverse: TTCCTGGAAAGGCACCTGAT. Lrg1 forward: TTGGCAGCATCAAGGAAGC, reverse: CAGATGGACAGTGTCGGCA. Ptgs2 forward: TTCAACACACTCTATCACTGGC, reverse: AGAAGCGTTTGCGGTACTCAT. Jmjd4 forward: AGCCTGGTGAGATGGTGTTTG, reverse: GCCATTGACCCAGTTGTGGT. GAPDH forward: CCTGGAGAAACCTGCCAAGTA, reverse: TCATACCAGGAAATGAGCTTGAC.

### Cell Culture and Treatment

Primary astrocyte culture was described as previously.^[^ ^31^ ^]^ The neonatal midbrain (P0‐3) was trypsinized and dissociated. Tissue was centrifuged for 5 min at 1000 rpm centrifugation, triturated, and resuspended in Dulbecco's modified Eagle's medium/Ham's F12 medium containing 10% fetal bovine serum (GIBCO, Gaithersburg, MD, USA). Cells were plated onto poly‐D‐lysine‐coated 6‐well plates to generate mixed glial cultures. Confluent mixed glial cultures were shaken at 220 rpm for 6 h at 37 °C to remove unwanted cell types (microglia, oligodendrocytes, neurons, and fibroblasts). The purity of astrocytes was >95% as determined with GFAP immunocytochemistry. Astrocytes were treated with α‐Syn preformed fibrils (PFF) (1 µg mL^−1^, ab218819, abcam) for 0.5, 2, 12 or 24 h. For pharmacological measurement, recombinant mouse LCN2 (1857‐LC‐050, R&D), 3‐Methyladenine (3‐MA, 10 mM, S2767, Selleck), MG‐132 (5 µM, S2619, Selleck) or Dynasore (80 µM, 304448‐55‐3, Sigma) was added to the cell culture medium for 30 min before α‐Syn PFF stimulation.

Co‐culture of neurons and astrocytes was carried out as previously described.^[^ ^32^ ^]^ The astrocytes were plated on the poly‐D‐lysine‐coated 24‐well plates and then were transfected with 24p3R plasmid or vector for 24 h. The culture medium was then replaced with neuron culture medium for 24 h. Neurons were obtained from the ventral mesencephalic tissues of C57BL/6 mice on embryonic day 14/15 (E14/15). Briefly, mesencephalic cells were dissociated by trypsinization (0.25% trypsin and 0.02% EDTA in Ca^2+^‐ and Mg^2+^‐free Hanks’ balanced salt solution) at 37 °C for 10 min, followed by gentle triturating in plating medium (h‐DMEM supplemented with 10% fetal bovine serum and 10% horse serum). Cells were seeded onto the above 24‐well plates containing astrocytes in plating medium and allowed to adhere for 4 h. After cell adherence, the medium was replaced by neurobasal medium supplemented with 2% B‐27 (Gibco‐BRL) and 0.5 mM l‐glutamine (Sigma). Half‐media changes were performed every 3.5 days. Neuron and astrocyte were co‐cultured and treated with α‐Syn PFF for 10 days before further testing.

### Cell Transfection

For Lcn2 or 24p3R plasmid transfection, astrocytes were transfected with plasmids expressing Flag‐Lcn2, Flag‐24p3R (Hanbio Biotechnology Co., Ltd., Shanghai, China) or Flag‐24p3R deletion mutant (deleted AA153‐183) in OPTI‐MEM‐reduced serum medium (Gibco, USA) using lipofectamine 3000 reagent (Invitrogen, Life Technologies) for 48 h. For knockdown of 24p3R, the siRNA targeting 24p3R (siRNA‐1023, sense: GGCGAUUUCUACAGCGAAUTT; antisense: AUUCGCUGUAGAAAUCGCCTT. siRNA‐1316, sense: CUGGGCUUCACCAACUUUATT; antisense: UAAAGUUGGUGAAGCCCAGTT) (Genepharma, Shanghai, China) was transfected into astrocytes using lipofectamine 3000 reagent for 48 h before stimulation according to the instructions provided.

### Protein Labeling

Commercial recombinant proteins α‐Syn PFF were labeled with Atto 488 (Sigma–Aldrich, 38 371) according to the manufacturer's instructions. After labeling, 100 mM glycine was added to quench the reaction and the proteins were subjected to Amicon Ultra‐0.5 mL Centrifugal Filters to remove any unreacted label. The fluorescently labeled α‐Syn PFF were sonicated before incubating with cells.

### Flow Cytometry Analysis

After the treatment, astrocytes were treated with 1 µg mL^−1^ of Atto 488‐labeled α‐Syn PFF for 2 h at 37 °C. These cells were washed twice with phosphate‐buffered saline (PBS) and trypsinized for 5 min at 37 °C. The cells were collected and analyzed using a Flow Cytometer (Guava Easy Cyte8, Millipore, USA). Total of 5000 events were recorded per sample. Flow data were analyzed with the FCS Express software.

### Surface Plasmon Resonance Analysis

To study the interaction between α‐Syn and 24p3R, the surface plasmon resonance (SPR) was performed using a Reichert 4SPR instrument (Reichert, USA) as previously described.^[^ ^33^ ^]^ Recombinant mouse 24p3R protein was purchased from CUSBIO (#CSB‐EP887559MO). Different concentrations of α‐Syn PFF (ab218819, abcam) were run over SPR with the CM5 chip (GE, USA) using the running buffer containing 1.8 mmol/l KH2PO4, 10 mmol L^−1^ Na2HPO4, 137 mmol L^−1^ NaCl, 2.7 mmol L^−1^ KCl, and 0.005% Tween‐20 (pH 7.8). The binding and dissociation rates were measured at a flow rate of 25 µl min^−1^. The injection of the ligands was performed for 1.5 min followed by a flow with ligand‐free buffer to analyze the dissociation for 2.5 min. Curves were corrected for nonspecific ligand binding by subtracting the signal obtained for the negative control flow cell. The equilibrium dissociation constant (Kd) was derived from a simple 1:1 interaction model using the Reichert data evaluation software.

### Proximity Ligation Assay

As previously described,^[^ ^34^ ^]^ 24p3R‐ α‐Syn interaction in astrocytes was detected using the Duolink Proximity Ligation Assay (PLA) assay kit (Sigma–Aldrich, DUO92101) following the manufacturer's protocol. After treatments, cells on slides were fixed with 4% paraformaldehyde and permeabilized with 0.3% Triton X‐100 in PBS. After blocking at 37 °C for 1 h, the slides were incubated with mouse primary α‐Syn antibody (610 786, BD) and rabbit primary 24p3R antibody (SAB3500306, Sigma–Aldrich) at 4 °C overnight and then with PLA probe solution for 1 h at 37 °C. After being washed, the slides were incubated for 30 min at 37 °C and then incubated with the amplification solution at 37 °C for 100 min protected from light. Finally, cell nuclei were stained with DAPI (Invitrogen, D1306), and the slides were imaged using the confocal laser scanning microscopy platform Leica TCS SP8.

### Sequential α‐Synuclein Extraction

Brain tissues were homogenized in Triton lysis buffer (1% Triton X‐100 in 50 mM Tris, 150 mM NaCl, pH 7.6) containing phosphatase and protease inhibitor cocktail, sonicated, and centrifuged at 100 000 g for 60 min at 4 °C.). Pellets were washed once in Triton lysis buffer, resuspended into SDS lysis buffer (2% SDS in 50 mM Tris, 150 mM NaCl, pH7.6) and centrifuged at 100 000 g for 30 min at 22 °C. Supernatants from Triton X‐100 and SDS extractions were collected for WB analysis.

### Iron and Malondialdehyde Measurement

The concentration of malondialdehyde (MDA) and iron content was measured using a commercially available kits (A003‐4‐1, A039‐2‐1, Nanjing Jiancheng Bioengineering Institute, China) according to the manufacturer's instructions.

### WB Analysis and Co‐Immunoprecipitation (co‐IP)

Brain tissues and cells were homogenized in RIPA lysis buffer. The protein was separated and transferred onto PVDF membranes (IPVH00010, Millipore, Billerica, MA, USA). Membranes were incubated with following primary antibodies overnight at 4 °C. Immuno‐reactive bands were detected by ImageQuant LAS 4000 imaging system (GE Healthcare, Pittsburgh, PA, USA) and quantified using ImageJ software. The following primary antibodies were used: anti‐LCN2 (AF1757, R&D), anti‐24p3R (SAB3500306, Sigma‐Aldrich), anti‐p‐α‐Syn (Ab51253, Abcam), anti‐α‐Syn (610 786, BD), anti‐TH (MAB318, 1:1000, Millipore, Billerica, MA, USA), anti‐β‐actin (BM0627, Boster, Pleasanton, CA, USA).

For co‐IP, astrocytes were lysed, centrifuged and the supernatants were incubated with anti‐α‐Syn (610 786, BD), anti‐β‐Amyloid (803 001, BioLegend), anti‐LCN2 (AF1757, R&D) or anti‐24p3R (SAB3500306, Sigma–Aldrich) antibodies at 4 °C overnight, and precipitated with protein A/G‐agarose beads (sc‐2003, Santa Cruz, CA, USA) for 4 h at 4 °C. The immunoprecipitated proteins were analyzed by WB analysis.

HEK‐293T cells in six‐well plates were transfected with mouse Flag‐24p3R plasmids, Flag‐24p3R D1 deletion mutant (deleted AA123‐401) plasmids, Flag‐24p3R D2 deletion mutant (deleted AA123‐183) plasmids, Flag‐24p3R D3 deletion mutant (deleted AA123‐152) plasmids or Flag‐24p3R D4 deletion mutant (deleted AA153‐183) plasmids for 24 h and then were treated with α‐Syn for 2 h. After that, HEK‐293T cells were lysed, centrifuged and the supernatants were incubated with anti‐α‐Syn (610 786, BD) antibodies at 4 °C overnight, and precipitated with protein A/G‐agarose beads (sc‐2003, Santa Cruz, CA, USA) for 4 h at 4 °C. The immunoprecipitated proteins were analyzed by WB analysis. Full‐length Western Blots were available on Figure S12 (Supporting Information).

### Statistical Analysis

All data were analyzed using GraphPad Prism 9 software. All results were shown as means ± SEM. One way or two‐way analysis of variance with the Tukey's post hoc test was used for comparison among different treatments and genotypes and Student's t test was used to assess the differences between two groups. The results were considered significant at p<0.05

## Conflict of Interest

The authors declare no conflict of interest.

## Author Contributions

Y.‐Y.J. and T.T. contributed equally to this work. M.L. conceived and designed the study. R.‐H.D. designed the study and wrote the paper. Y.‐Y.J., T.T., Z.Z., R.‐A.W., Z.‐Y.Z., Y.L., L.C., K.‐Z.Z., and C.W. performed the experiments and analyzed the data. G.H., W.‐W.Y., X.L and Y.‐M.L. revised the paper. All authors read and approved the final manuscript.

## Supporting information



Supporting Information

Supporting Information

## Data Availability

The data that support the findings of this study are available in the supplementary material of this article.
