# Supplementary material for: Extracellular LCN2 Binding to 24p3R in Astrocytes Impedes α‐Synuclein Endocytosis in Parkinson's Disease
Source: Adv Sci (Weinh). 2025 Jul 21;12(39):e01694. doi: 10.1002/advs.202501694 (PMC12533313; doi:10.1002/advs.202501694)
Supplement: Supplementary file 1 — Supporting Information [file ADVS-12-e01694-s001.docx]

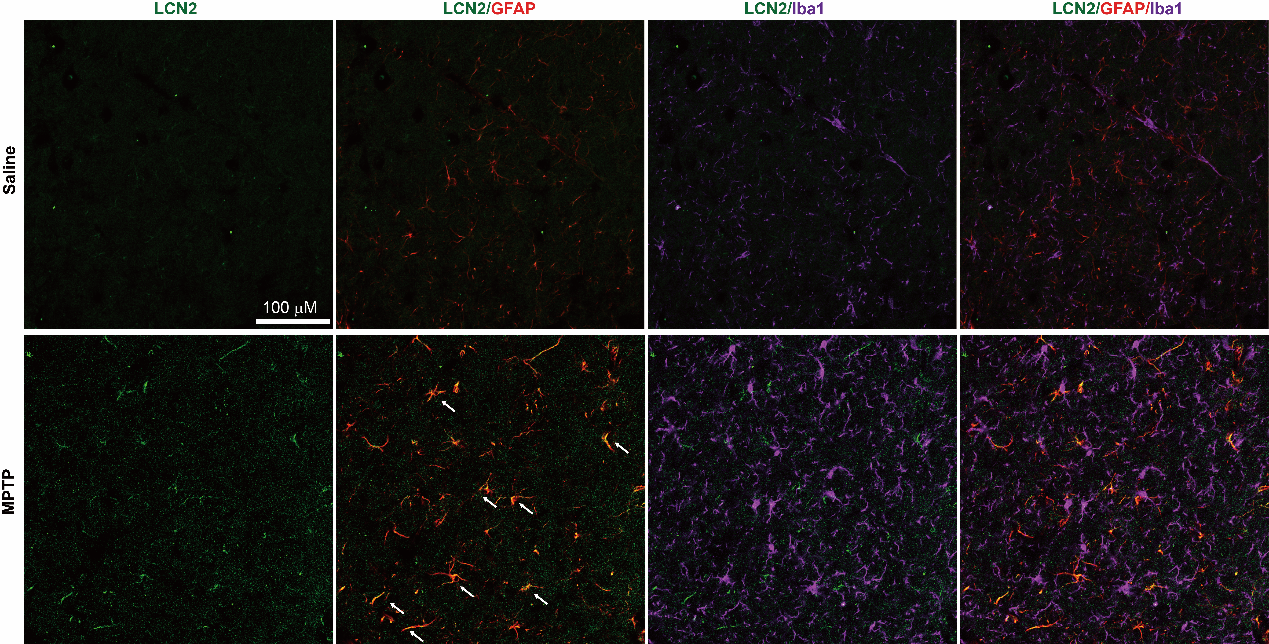


**Figure S1** **LCN2 is increased in astrocytes of MPTP-treated mice.** LCN2 immunohistochemical signal in astrocytes and microglia from the SNpc of MPTP-treated mice after tyramide signal amplification.


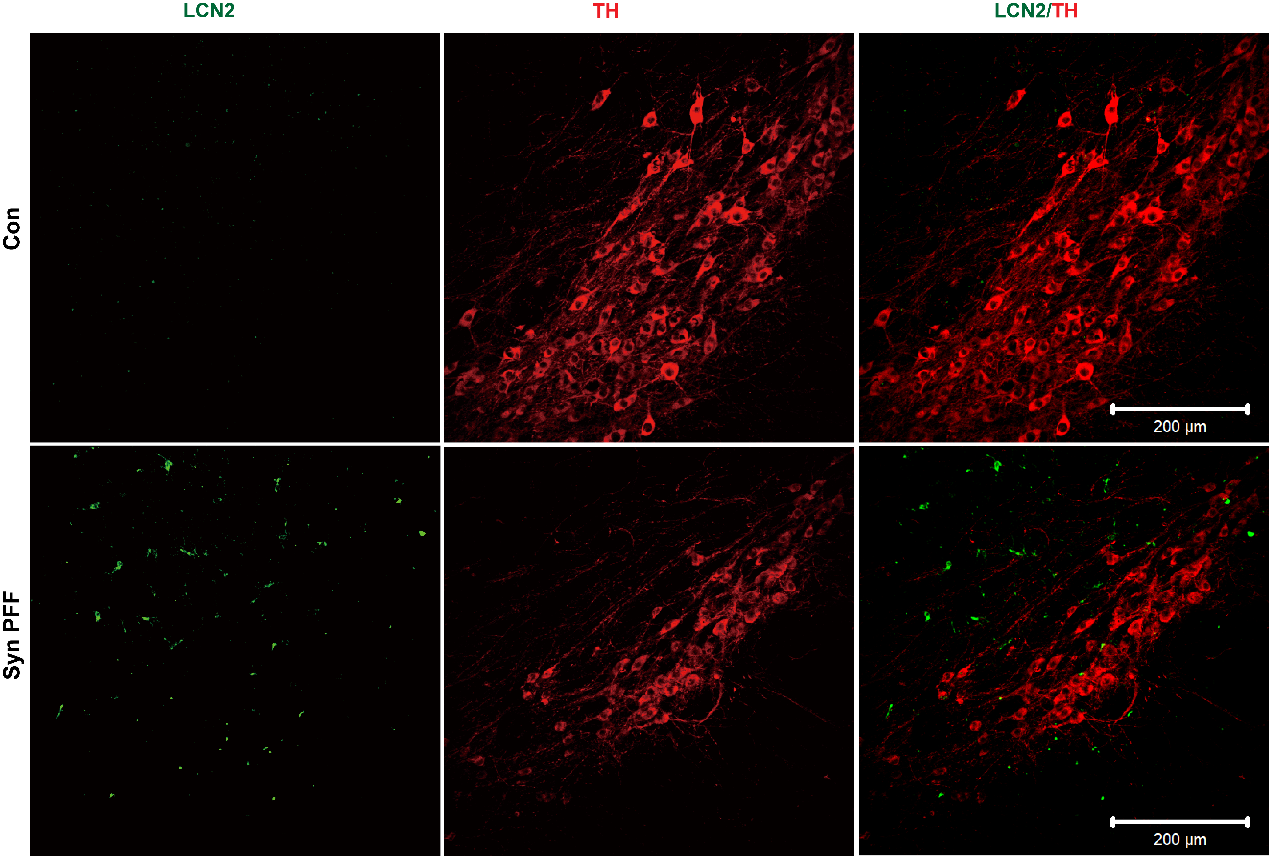


**Figure S2** **LCN2 is not expressed in TH^+^-neuron.** Representative double-immunostaining for LCN2 (green) and TH (red) in the SNpc of α-Syn PFF-injected PD mice.


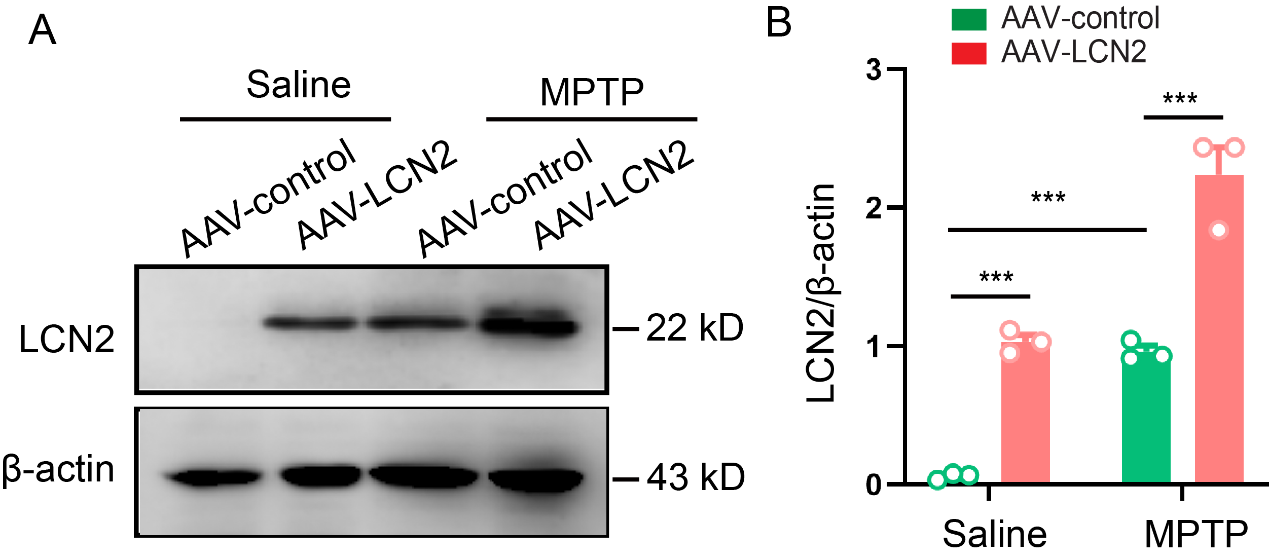


**Figure S3** **LCN2 expression is increased in the SNpc of AAV-LCN2-injected mice.** A-B) Western blot analysis of LCN2 expression in the SNpc of AAV-control or AAV-LCN2-injected mice (n=3 animals). The data shown are the mean ± SEM. Two-way ANOVA with Tukey’s post-hoc test was used. ^***^p<0.001.


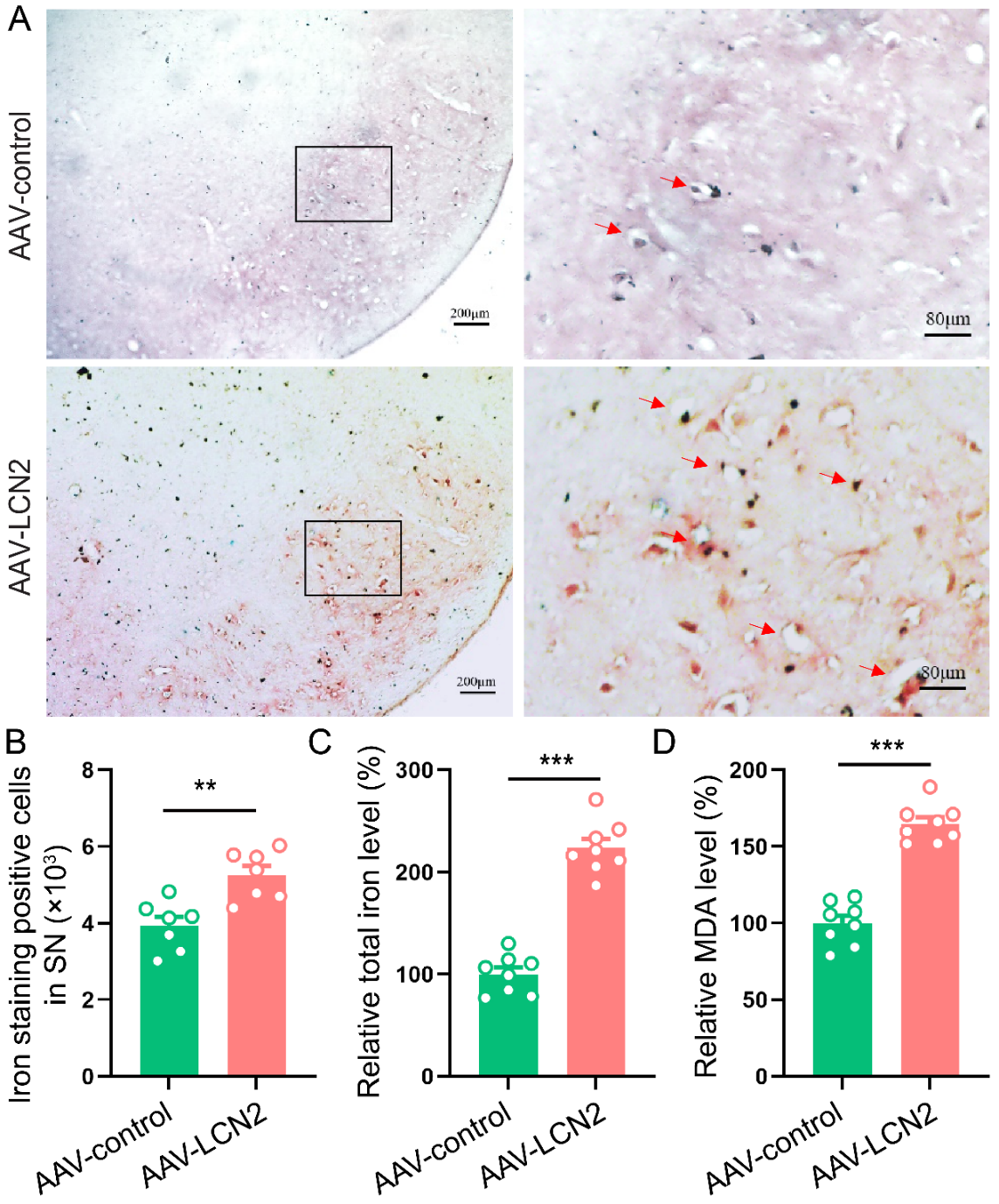


**Figure S4 Increased iron levels in the** **midbrain of astrocytic LCN2-overexpressed mice.** A) Perls’iron staining in the midbrain of AAV-control and AAV-LCN2-injected mice. B) Quantification of iron staining positive cells (n=7 animals for each group). C-D) Relative total iron level and MDA level in the midbrain (n=8 animals for each group). The data shown are the mean ± SEM. Unpaired t test was used. ^**^p<0.01, ^***^p<0.001.


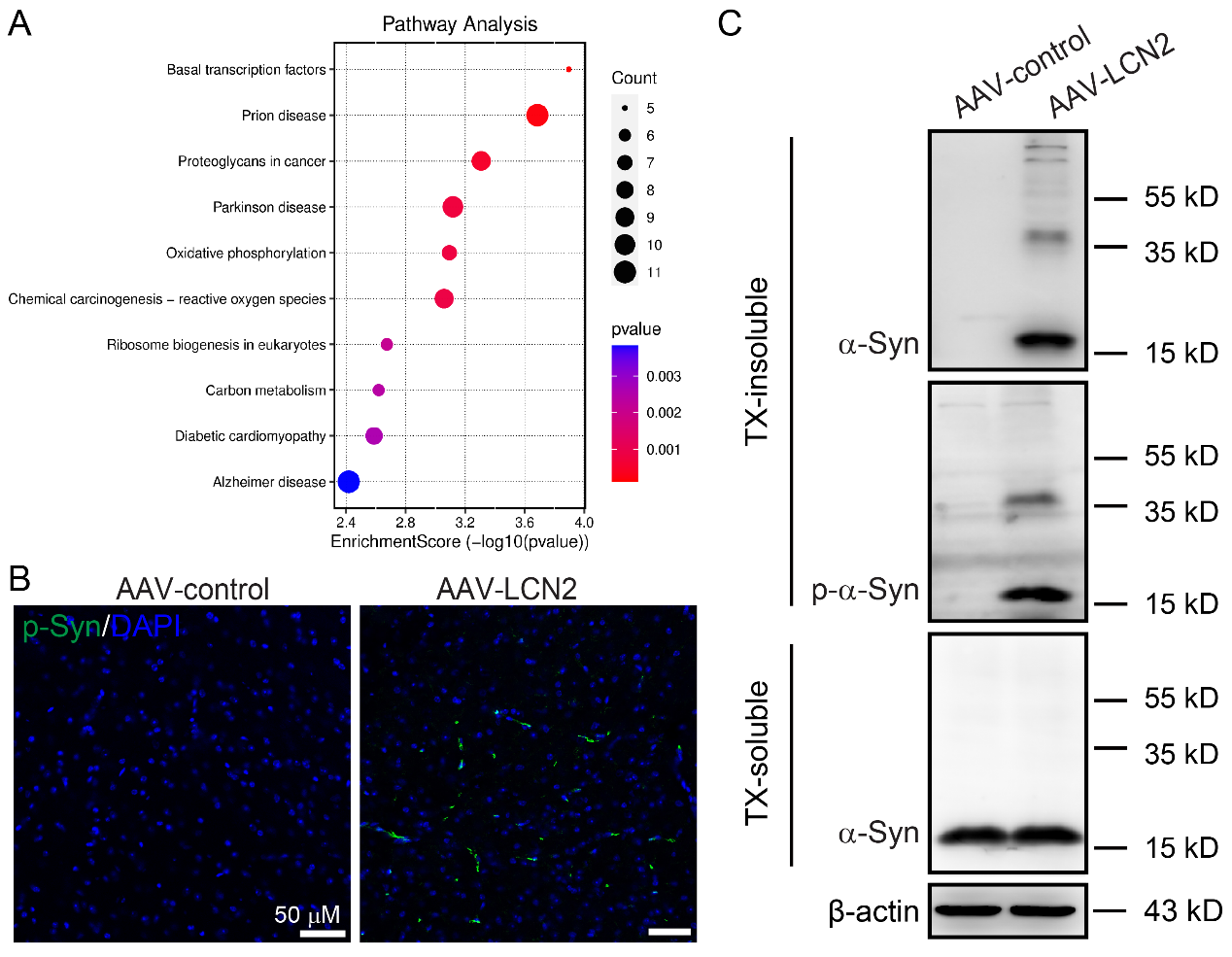


**Figure S5** **Astrocytic LCN2 overexpression aggravates α-Syn accumulation in mice.** A) Top 10 KEGG pathways enrichment analysis of potential targets for LCN2 by MS. B) Representative immunostaining for p-Syn (green) in the SNpc of astrocytic LCN2-overexpressed mice. DAPI stains nucleus (blue). C) Western blot analysis of TX-insoluble or TX-soluble α-Syn and p-α-Syn in the SNpc of astrocytic LCN2-overexpressed mice.


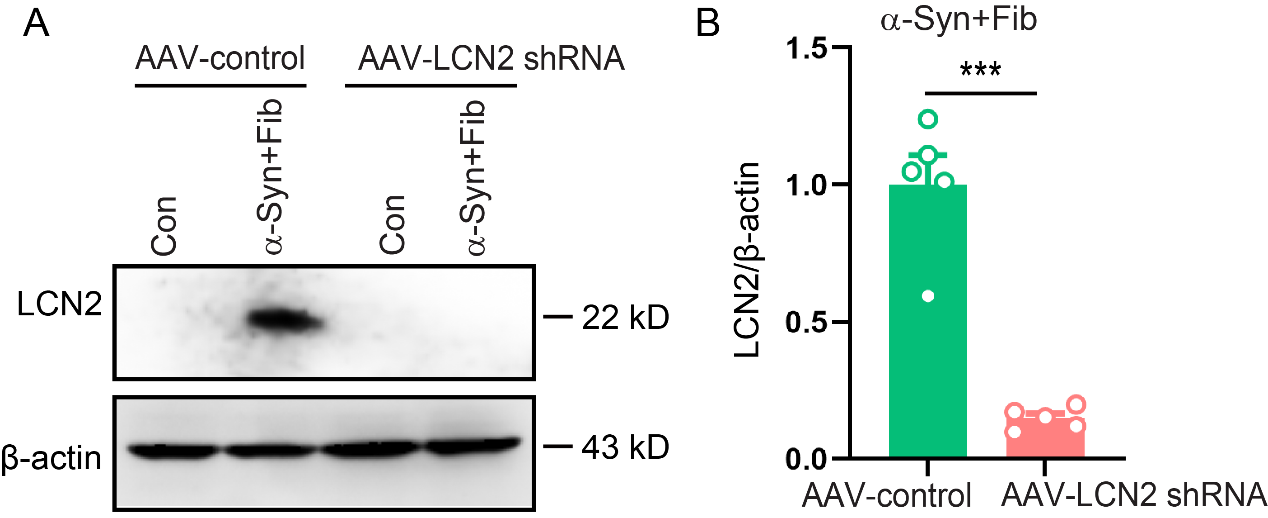


**Figure S6** **LCN2 expression is reduced in the SNpc of AAV-mediated LCN2 shRNA-injected mice.** A-B) Western blot analysis of LCN2 expression in the SNpc of AAV-control or AAV-LCN2 shRNA-injected mice (n=5 animals). The data shown are the mean ± SEM. Unpaired t test was used. ^***^p<0.001.


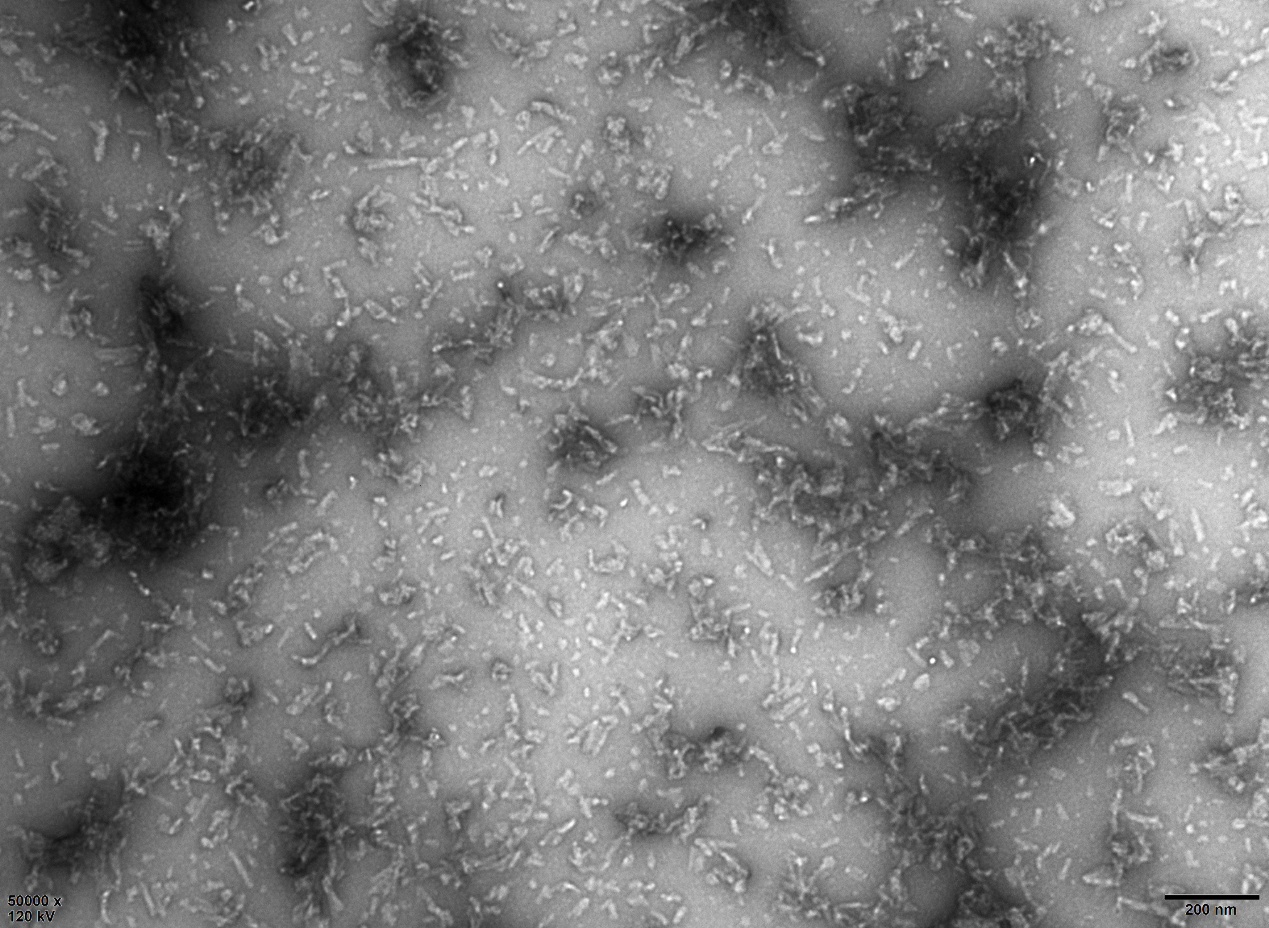


**Figure S7** **Transmission electron microscopy image showing the structure of sonicated α-Syn preformed fbril (PFF).** The average PFF length was 47.32 ± 2.39 nm after sonication.


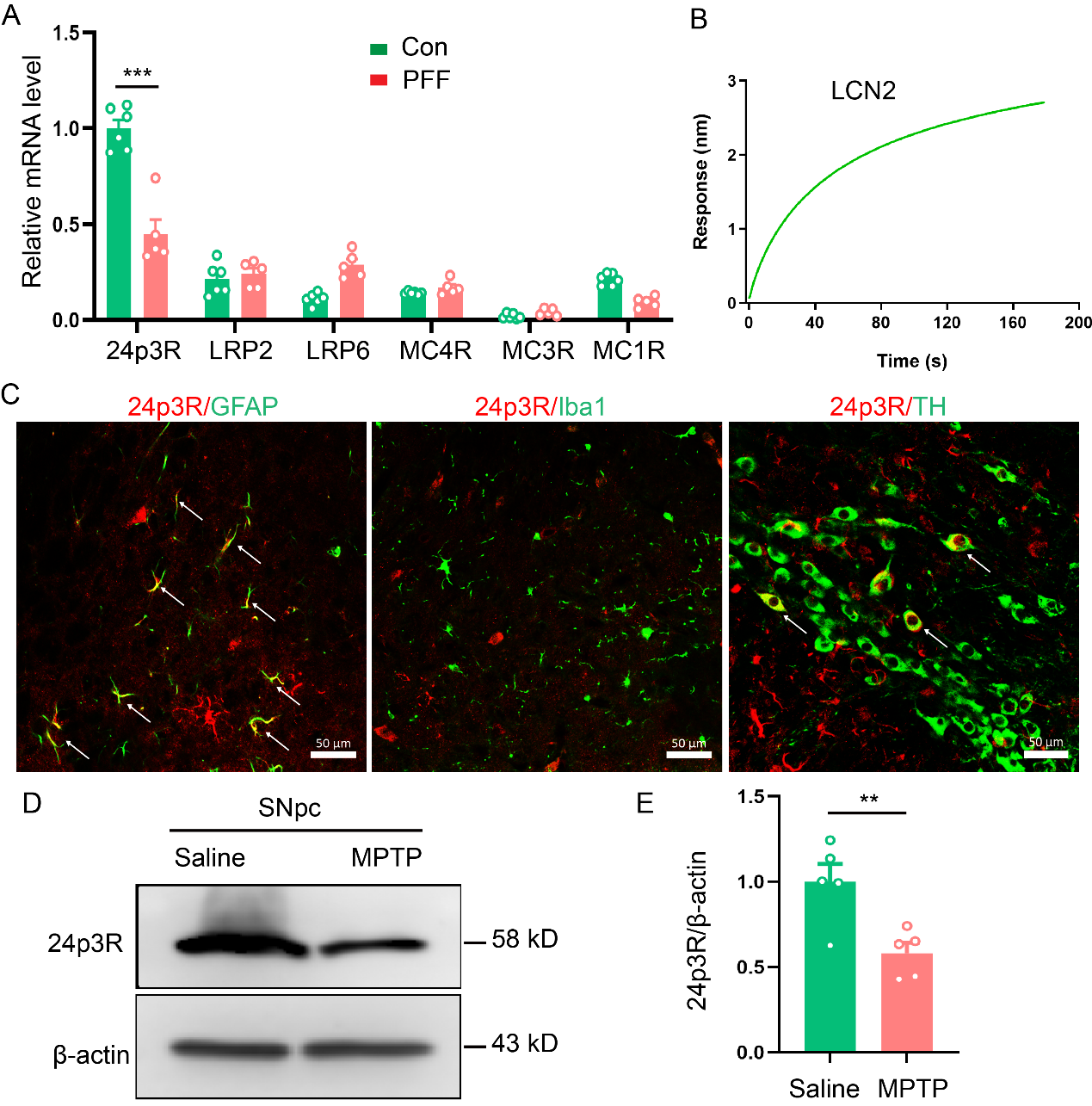


**Figure S8** **24p3R is the primary LCN2 receptor in astrocytes.** A) qPCR analysis measuring the mRNA levels of indicated genes in astrocytes treated with α-Syn PFF (six independent experiments). B) SPR assay to evaluate the affinity between LCN2 and purified mouse 24p3R protein. C) 24p3R immunohistochemical signal in astrocytes, microglia and TH neuron in the SNpc. D-E) Western blot analysis of 24p3R expression in the SNpc of MPTP-treated mice (n=5 animals). The data shown are the mean ± SEM. Unpaired t test was used. ^**^p<0.01, ^***^p<0.001.


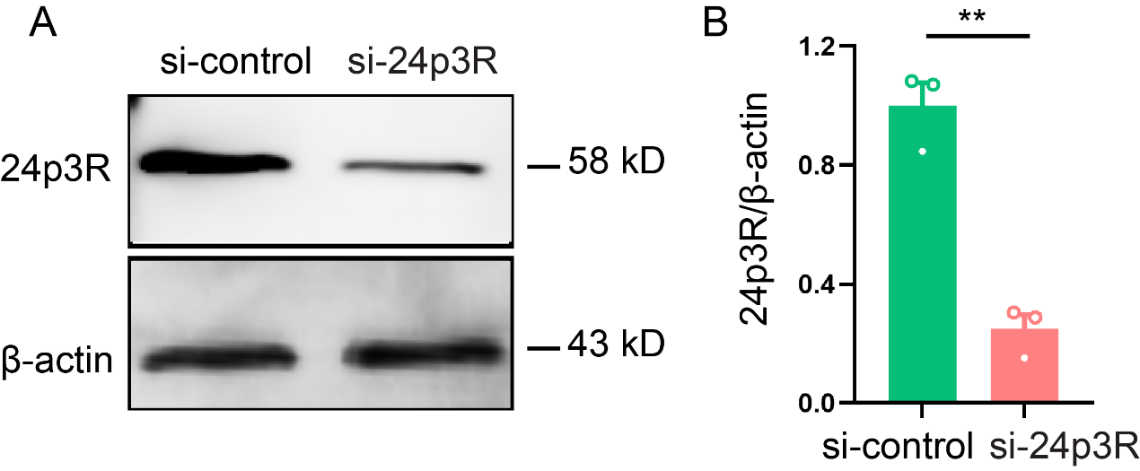


**Figure S9** **24p3R expression is reduced in astrocytes by siRNA-mediated 24p3R silencing.** A-B) Western blot analysis of 24p3R expression in astrocytes transfected with control siRNA (si-control) or 24p3R siRNA (si-24p3R) (Three independent experiments). The data shown are the mean ± SEM. Unpaired t test was used. ^**^p<0. 01.


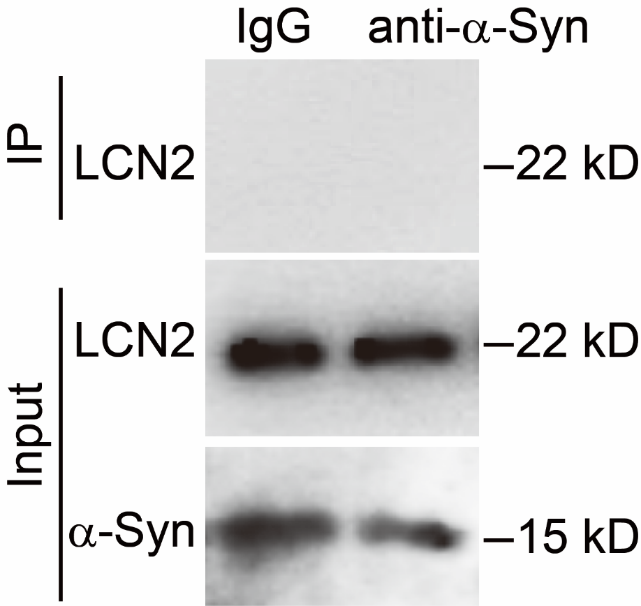


**Figure S10** **LCN2 fails to bind to α-Syn in the supernatants of astrocytes.** Astrocytes were transfected with LCN2 plasmids for 48 h and were then stimulated with α-Syn PFF (1 μg/ml) for 2 h. Immunoprecipitation and immunoblot analysis of the interaction of LCN2 with α-Syn in the supernatants of astrocytes using anti-IgG or anti-α-Syn antibodies.


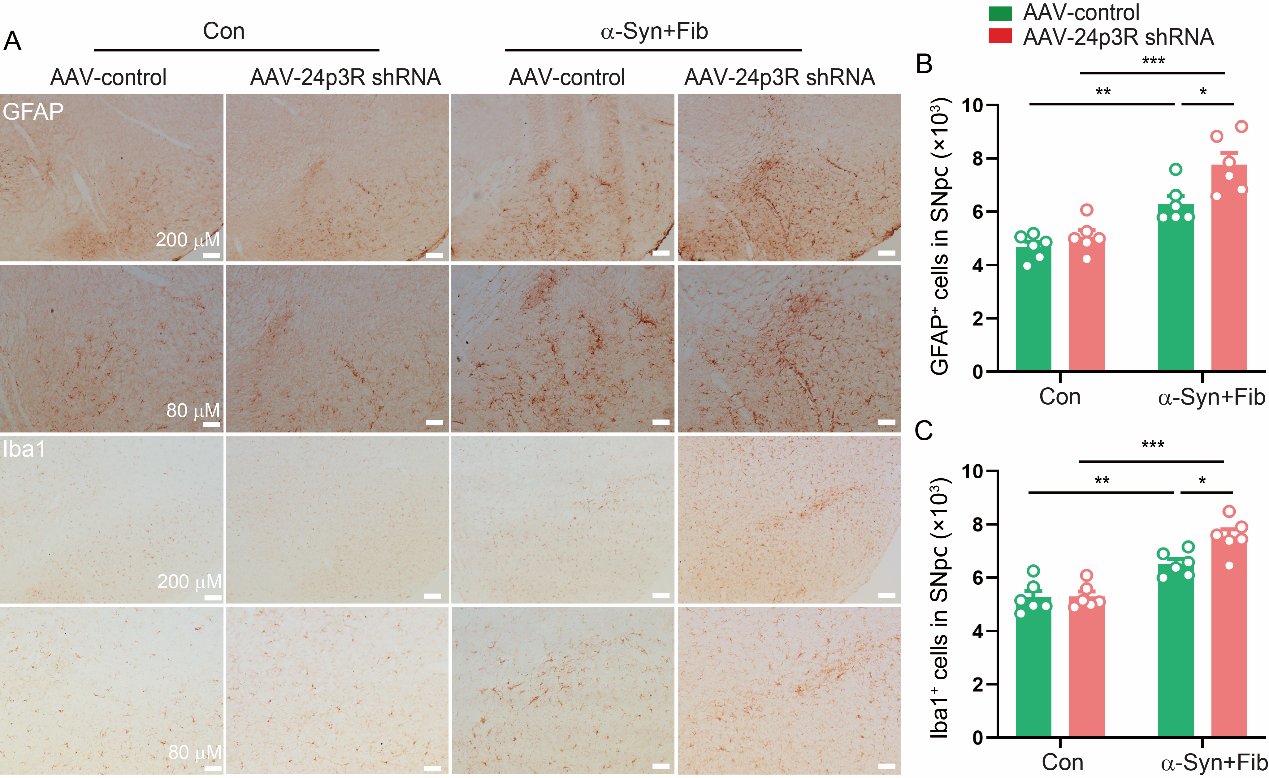


**Figure S11** **Astrocytic 24p3R deletion promotes the activation of astrocytes and microglia in α-Syn PFF-injected mice.** A) Microphotographs of GFAP-positive astrocytes and Iba1-positive microglia in the SNpc. B-C) Stereological counts of GFAP-positive astrocytes (B) and Iba1-positive microglia (C) in the SNpc (n=6 animals for each group). The data shown are the mean ± SEM. Two-way ANOVA with Tukey’s post-hoc test was used. ^*^p<0.05, ^**^p<0.01, ^***^p<0.001.


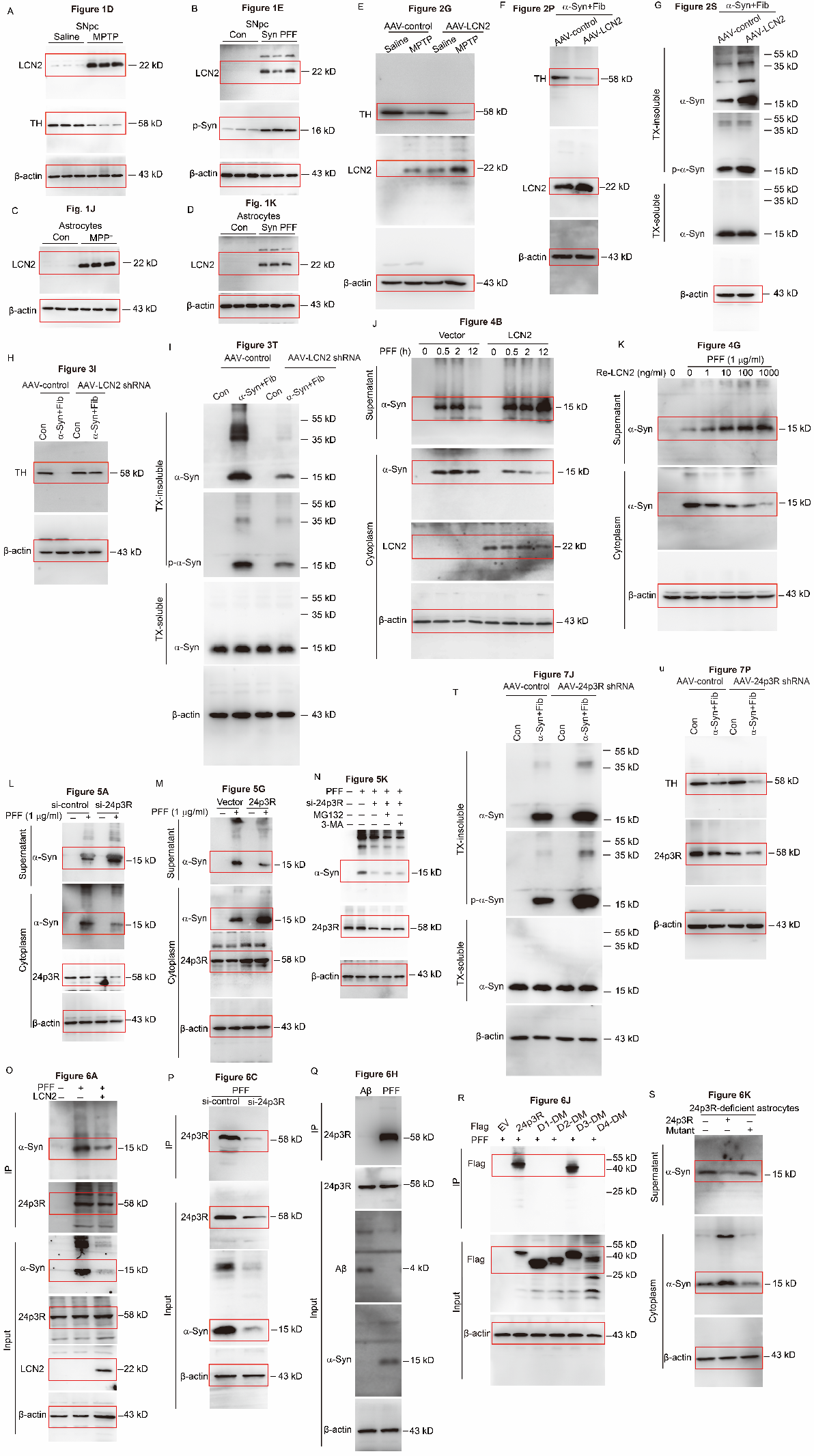


**Figure S12 Full-length blots of cropped blots.**

A) Full-length Western blot for LCN2 (top), TH (middle) and β-actin (bottom) in Figure 1D. B) Full-length Western blot for LCN2 (top), p-Syn (middle) and β-actin (bottom) in Figure 1E. C-D) Full-length Western blot for LCN2 (top) and β-actin (bottom) in Figure 1J and in Figure 1K. E-F) Full-length Western blot for TH (top), LCN2 (middle) and β-actin (bottom) in Figure 2G and in Figure 2P. G) Full-length Western blot for TX-insoluble α-Syn and p-Syn (top), TX-soluble α-Syn (middle) and β-actin (bottom) in Figure 2S. H) Full-length Western blot for TH (top) and β-actin (bottom) in Figure 3I. I) Full-length Western blot for TX-insoluble α-Syn and p-Syn (top), TX-soluble α-Syn (middle) and β-actin (bottom) in Figure 3T. J) Full-length Western blot for α-Syn in supernatants (top) and α-Syn, LCN2, and β-actin in cytoplasm (bottom) in Figure 4B. K) Full-length Western blot for α-Syn in supernatants (top) and α-Syn and β-actin in cytoplasm (bottom) in Figure 4G. L-M) Full-length Western blot for α-Syn in supernatants (top) and α-Syn, 24p3R, and β-actin in cytoplasm (bottom) in Figure 5A and in Figure 5G. N) Full-length Western blot for α-Syn (top), 24p3R (middle) and β-actin (bottom) in Figure 5K. O) Full-length Western blot for α-Syn and 24p3R (IP, top) and α-Syn, 24p3R, LCN2, and β-actin (input, bottom) in Figure 6A. P) Full-length Western blot for 24p3R (IP, top) and 24p3R, α-Syn, and β-actin (input, bottom) in Figure 6C. Q) Full-length Western blot for 24p3R (IP, top) and 24p3R, Aβ, α-Syn, and β-actin (input, bottom) in Figure 6H. R) Full-length Western blot for Flag (IP, top) and Flag and β-actin (input, bottom) in Figure 6J. S) Full-length Western blot for α-Syn in supernatants (top) and α-Syn and β-actin in cytoplasm (bottom) in Figure 6K. T) Full-length Western blot for TX-insoluble α-Syn and p-Syn (top), TX-soluble α-Syn (middle) and β-actin (bottom) in Figure 7J. U) Full-length Western blot for TH (top), 24p3R (middle) and β-actin (bottom) in Figure 7P.
